# Generation of fully functional hepatocyte-like organoids from human induced pluripotent stem cells mixed with Endothelial Cells

**DOI:** 10.1038/s41598-019-45514-3

**Published:** 2019-06-20

**Authors:** Giuseppe Pettinato, Sylvain Lehoux, Rajesh Ramanathan, Mohamed M. Salem, Li-Xia He, Oluwatoyosi Muse, Robert Flaumenhaft, Melissa T. Thompson, Emily A. Rouse, Richard D. Cummings, Xuejun Wen, Robert A. Fisher

**Affiliations:** 1 Department of Surgery, Beth Israel Deaconess Medical Center, Harvard Medical School, Boston, MA USA; 2 Glycomics Core, Beth Israel Deaconess Medical Center, Harvard Medical School, Boston, MA USA; 3 Department of Surgery, University of Pittsburgh Medical Center, Pittsburgh, PA USA; 4 Department of Chemical and Life Science Engineering, Virginia Commonwealth University, Richmond, VA USA; 5 Neurosurgical Service, Beth Israel Deaconess Medical Center, Harvard Medical School, Boston, MA USA; 6 Department of Medicine, Beth Israel Deaconess Medical Center, Harvard Medical School, Boston, MA USA

**Keywords:** Regeneration, Stem-cell biotechnology, Induced pluripotent stem cells

## Abstract

Despite advances in stem cell research, cell transplantation therapy for liver failure is impeded by a shortage of human primary hepatocytes (HPH), along with current differentiation protocol limitations. Several studies have examined the concept of co-culture of human induced pluripotent cells (hiPSCs) with various types of supporting non-parenchymal cells to attain a higher differentiation yield and to improve hepatocyte-like cell functions both *in vitro* and *in vivo*. Co-culturing hiPSCs with human endothelial cells (hECs) is a relatively new technique that requires more detailed studies. Using our 3D human embryoid bodies (hEBs) formation technology, we interlaced Human Adipose Microvascular Endothelial Cells (HAMEC) with hiPSCs, leading to a higher differentiation yield and notable improvements across a wide range of hepatic functions. We conducted a comprehensive gene and protein secretion analysis of our HLCs coagulation factors profile, showing promising results in comparison with HPH. Furthermore, a stage-specific glycomic analysis revealed that the differentiated hepatocyte-like clusters (HLCs) resemble the glycan features of a mature tissue rather than cells in culture. We tested our HLCs in animal models, where the presence of HAMEC in the clusters showed a consistently better performance compared to the hiPSCs only group in regard to persistent albumin secretion post-transplantation.

## Introduction

End stage liver disease represents a potentially fatal condition. Currently treatment options are limited to liver transplantation, which is restricted by shortage of liver donors, and carries high perioperative risks and the necessity of lifelong immunosuppression^1–6^. HPH transplantation and bio-artificial liver (BAL) devices have been used as temporary alternatives^7^. However, the loss of function of HPH with storage and recovery from cryopreservation hinder their efficacy^8–12^.

Human induced pluripotent stem cells (hiPSCs) represent a valid alternative in regenerative medicine providing a potentially inexhaustible source of autologous stem cells that can be differentiated into numerous tissues, including hepatocyte-like cells (HLCs), enabling the possibility of homologous hepatocyte transplantation, with the potential of limiting immune rejection^13–21^. Nevertheless, currently available protocols are still limited by low scalability, residual immature genotypes after differentiation, and poor long-term cell function after transplantation^22,23^.

Various hepatic differentiation strategies using hiPSCs have been described^24–29^. We have demonstrated that embryoid bodies (EBs) from hiPSCs represent promising alternatives, as they are 3D clusters with the potential of three-germ layer generation, and showing hepatic *in vitro* lineage-specific differentiation through Wnt/β-catenin pathway inhibition, that mimic the *in vivo* hepatogenesis observed in embryos^21^, yet, avoiding the use of ROCK inhibitor for their fabrication^30,31^. Exclusion of the xeno-factors will enable less safety precautions in translation to cell therapy^32^.

Engraftment and long-term function of transplanted cell clusters is historically impaired due to: nidation failure; cell destruction caused by inflammatory mediated destruction and/or apoptosis; and environmental factors such as hypoxia, and long term immune recognition. Improving engraftment and promoting neovascularization, is the first step to cell survival and long-term HLCs function. Human endothelial cells (hECs) constitute 19% of the total adult liver cell mass and may also promote hepatic differentiation. The incorporation of hECs with hiPSCs within hEBs could provide a sustained hepatocyte function *in vivo*. We previously explored the use of hECs interlaced with hiPSCs to ameliorate the challenges of engraftment of the transplanted cells^33^. The ability of hECs to secrete endogenous angiogenic factors, which might aid in recruiting new blood vessels to the transplantation site, has been described^34–36^.

Understanding the biology of the cell surface glycomic structure in differentiated HLCs and this relationship to immune recognition and cell to cell interactions has not been fully addressed^37^. Here we have analyzed the post-translational glycosylation of proteins of our differentiated HLCs, and compared them with HPH, for the potential development of new glycan-based tools to study HLCs differentiation and transplantation patterns of host integration.

Coagulation factor, expression and secretion from differentiated human HLCs, is another important function that has not been explored in depth in previous publications^38–41^. Gene expression and protein secretion of the major components of the coagulation cascade, both intrinsic and extrinsic, were studied to assess the capability of our differentiated HLCs to contribute to physiologic hemostasis.

In the present study, we explored the ability of Human Adipose Microvascular Endothelial Cells (HAMEC) interlaced with hiPSCs to enhance the differentiation of hiPSCs derived HLCs. By using the 3D spheroid culture-based hepatic differentiation protocol, we created a multicellular product that contains both hiPSCs and HAMEC in the same cluster. This cell culture model, together with our already established hepatic differentiation protocol, allows the production of HLCs in large quantities for future transplantation therapy^21^.

## Results

### hiPSC-EBs were differentiated in 3D culture with and without HAMEC into HLCs

Stage-specific protein analysis by immunofluorescence was performed at the end of each stage to ensure the stepwise differentiation of the hiPSC-EBs with and without HAMEC. A progressive regulated pattern of stage-specific hepatic markers at the completion of each stage were observed in hiPSC-EB-HLCs and hiPSC-EB + EC-HLCs respectively (Supplementary Fig. 1a,b).

HLCs morphology showed a progressive increase in the clusters’ size from approximately 500 µm to 800–1,000 µm at the end of the differentiation process accompanied by a change in morphology (Supplementary Fig. 2a). HLCs were assessed for cell morphology. Light microscopy showed that the cells within the clusters exhibited the classical morphology of HPH, being polygonal in shape with enriched cytoplasmic granules (Supplementary Fig. 2b – Upper 10X; lower 20X)^24^. HLCs were also assessed for live/dead staining at the end of the differentiation protocol, and no core necrosis was observed (Supplementary Fig. 2c).

A ratio of 15% CD31 positive cells and 89% albumin positive cells was observed after terminal differentiation. In Supplementary Fig. 3 the top two panels show two representative optical cross-sections of our differentiated hiPSC-EB + EC-HLCs. In the lower panel is shown a representative image of the HLCs without HAMEC that did not display any CD31 positive cells as expected.

### HLCs with HAMEC had increased gene expression of coagulation factors, and prolonged duration of protein secretion

**Figure 1:**
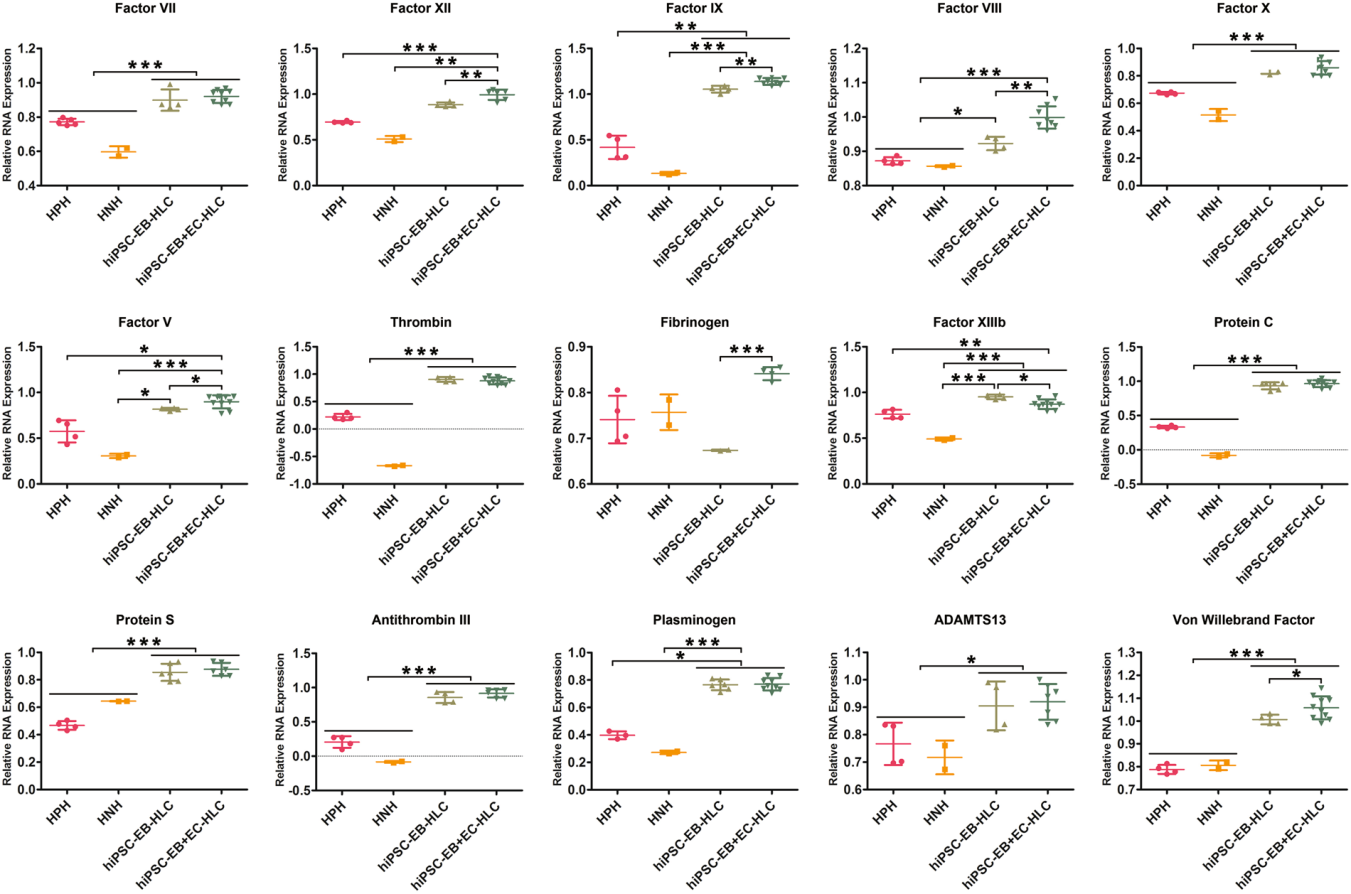
Real-time PCR of the coagulation cascade and fibrinolysis genes. The main factors involved in the coagulation cascade, from both the intrinsic, extrinsic and common pathway, as well as some factors involved in the fibrinolysis, were tested. In most of the cases, in both hiPSC-EB-HLCs and hiPSC-EB + EC-HLCs we observed a gene expression that was statistically higher than the one observed in human primary hepatocyte (HPH) and human neonatal hepatocyte (HNH). For some genes, such as FXII, FIX, FVIII, FV, Fibrinogen, FXIIIb, and VWF the expression of our differentiated hiPSC-EB + EC-HLCs was significantly higher than the hiPSC-EB-HLCs. HPH was used as a positive control for mature phenotype, while HNH was used as a control for immature genotype. Results for the differentiated hiPSCs for both conditions were normalized GAPDG and to undifferentiated hiPSCs (experimental negative control). Data presented as mean ± SD (n = 3). *p < 0.05; **p < 0.01; ***p < 0.001.

We hypothesized that interlacing hiPSC-EBs with HAMEC would synergistically improve function of hiPSC-EB-HLCs by the activating effect of HAMEC on HLCs. Following terminal differentiation of hiPSC-EB-HLCs with and without HAMEC, gene expression of most of the coagulation factors involved in the extrinsic, intrinsic, and common coagulation pathways, as well as factors involved in the thrombolysis and haemostasis processes were present in both hiPSC-EB-HLCs and hiPSC-EB + EC-HLCs (Fig. 1). Notably, Factor XII (p < 0.001), Factor IX (p < 0.001), Factor VIII (p < 0.001), Factor V (p < 0. 01), Fibrinogen (p < 0.0001), Factor XIIIb (p < 0. 01) and VWF (p < 0. 01), were statistically significant higher in hiPSC-EB + EC-HLCs when compared with hiPSC-EB-HLCs. When both hiPSC-EB + EC-HLCs and hiPSC-EB-HLCs were compared with the positive controls HPH and HNH, they showed a statistically higher genes expression in all the markers studied (p < 0. 01; p < 0. 001; p < 0. 0001).

**Figure 2:**
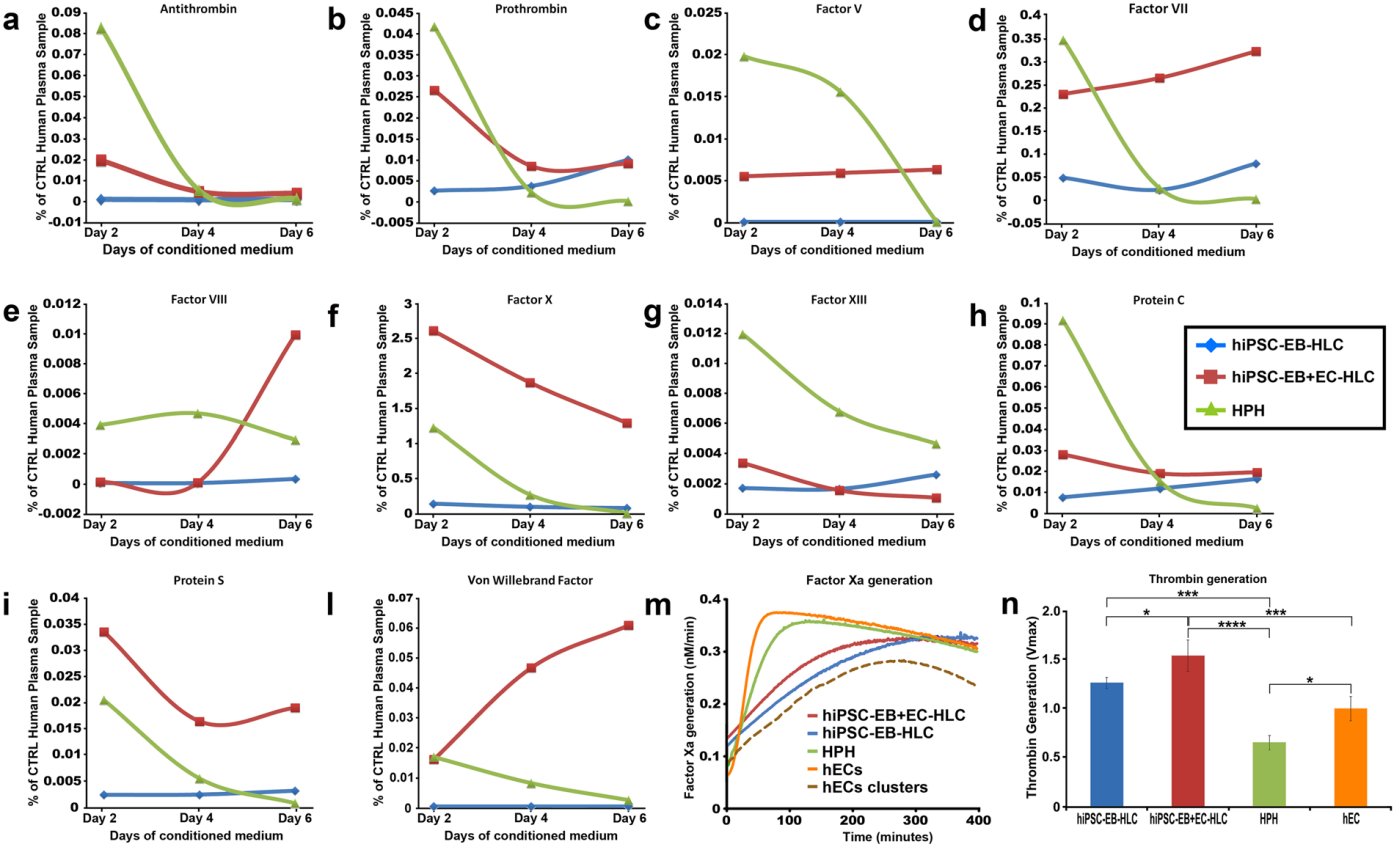
Quantification analysis of secreted coagulation factors. We collected the conditioned medium for 6 days every other day for both primary hepatocyte, hiPSC only and plus endothelial cells (HAMEC), and we analyzed the conditioned media to study the secretion of several proteins involved in the coagulation cascade. (**a**–**l**) As expected, primary hepatocyte showed higher level of coagulation factors secretion when compared with our experimental conditions in the beginning, however, starting from day four, they start to lose functions, where our experimental conditions showed a better performance in most of the factors studied, and in certain cases even higher than the HPH themselves. In most of the cases hiPSC-EB + EC-HLCs performed better than hiPSC-EB-HLCs. (**m**) Factor Xa generation assay. The presence of endothelial cells in our differentiated clusters led to a higher affinity of the factor Xa generation enzyme to the substrate as indicated from the lower Km when comparing the hiPSC only with the hiPSCs plus endothelial cells. As positive control HPH, and HAMEC in monolayer culture and HAMEC in 3D culture (dashed line), were used. (**n**) Results for the thrombin generation assay showed that the microparticles released from hiPSC-EB + EC-HLCs amplified thrombin generation when compared with the condition without HAMEC.

To test the coagulation protein secretion, supernatants were analysed for 6 days in primary hepatocyte (HPH), hiPSC-EB-HLCs and hiPSC-EB + EC-HLCs. While HPH showed an initial higher level of coagulation factors secretion in comparison to our experimental conditions; they began to lose function on day 4, while hiPSC-EB + EC-HLCs showed persistent production of coagulation factors (Fig. 2a–l). VWF, and Factor VIII which are normally produced and secreted by the endothelial cells *in vivo*, were not present in our hiPSC-EB-HLCs, but both VWF and Factor VIII were observed in hiPSC-EB + EC-HLCs, indicating that HAMEC were functionally active within our liver organoids. The value for these factors increased over time until reaching a peak at day 6. Interestingly, HPH showed a moderate secretion of these factors, probably due to cross-contamination with sinusoidal cells during their isolation, but this secretion ended at day 4 and day 6 respectively of the observation time.

### Differentiated HLC plus HAMEC generated both Factor Xa and Thrombin

Analysis for the Factor Xa generation showed similar Vmax values for hiPSC-EB + EC-HLCs, hiPSC-EB-HLC, HPH, HAMEC and HAMEC clusters (Vmax: 0.3516 vs 0.3728 vs 0.3620 vs 0.3698 vs. 0.3104 respectively), indicating no major differences between the two experimental conditions plus or minus HAMEC (Fig. 2m). However, the Km values were markedly lower in the hiPSC-EB + EC-HLCs in comparison to the hiPSC-EB-HLCs (Km: 31.08 vs 55.44 respectively), suggesting that the presence of HAMEC led to a higher affinity of the factor Xa generating enzyme to its substrate (factor X). The considerably lower Km values for the HPH and HAMEC conditions in comparison to both experimental conditions with and without HAMEC might be due to HLCs 3D form, in contrast to the monolayer culture of HPH and HAMEC. This difference in configuration could potentially affect the enzyme affinity and subsequently the Km values (Fig. 2m). To address this hypothesis, we performed in parallel experiments using 3D clusters of HAMEC. The results obtained using the HAMEC 3D clusters showed a similar Km pattern of our differentiated HLCs plus and minus HAMEC, confirming our hypothesis (Km: 31.08 vs 55.44 vs 49.96 respectively) (Fig. 2m dashed line). Thrombin generation was also assessed in our differentiated HLC plus and minus HAMEC (Fig. 2n). As shown in Fig. 2n, the addition of HAMEC resulted in a statistically significant increase of the thrombin generation when compared with the condition without HAMEC (1.54 vs 1.26; p < 0.05) indicating that the micro-particles released from hiPSC-EB + EC-HLCs amplified thrombin generation. When related with HAMEC, hiPSC-EB + EC-HLCs showed a statistically significant higher value (1.54 vs 1; p < 0.0009). Thrombin generation efficiency of our differentiated HLCs plus HAMEC was also confirmed by a statistically higher value when compared with HPH (1.54 vs 0.65; p < 0.0001). Data were normalized to HAMEC and showed as fold increase (HAMEC normalized to 1).

### hiPSC-EB + EC-HLCs displayed greater hepatocyte gene and protein expression

**Figure 3:**
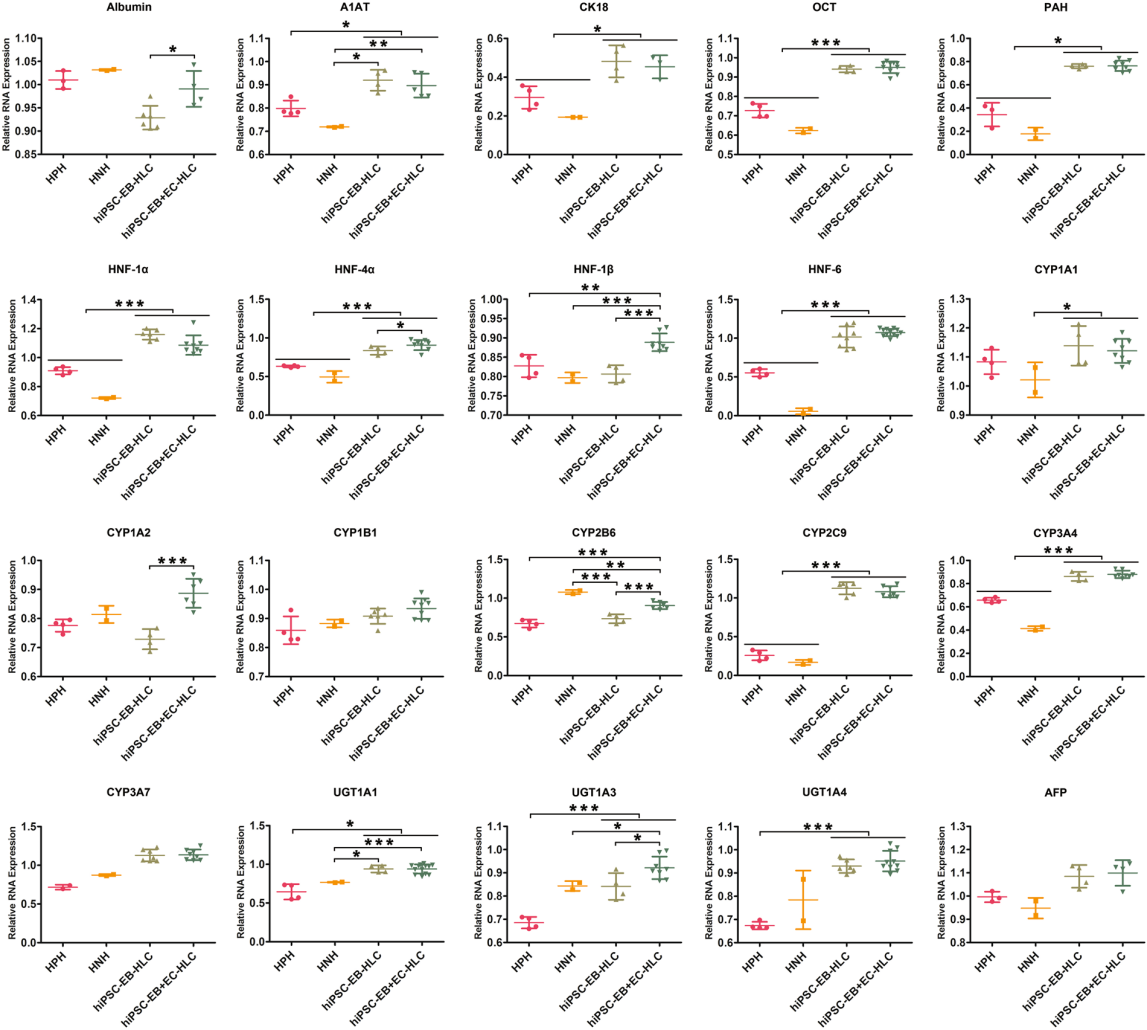
Tissue-specific gene expression analysis by Real-Time PCR. The relative quantities of tissue-specific genes were measured at the mRNA level to assess the final maturation of the terminally differentiated HLCs for both experimental conditions. The results showed an overall trend of higher expression of all markers for the hiPSC-EB + EC-HLCs when compared to the hiPSC-EB-HLCs, with statistically significant higher expression for the albumin, HNF-4α, HNF-4β, CYP1A2, CYP2B6 and UGT1A3. Almost for all the genes tested, both hiPSC-EB-HLCs and hiPSC-EB + EC-HLCs showed a gene expression that was statistically higher than the one observed in human primary hepatocyte (HPH) and human neonatal hepatocyte (HNH). HPH was used as a positive control for mature phenotype, while HNH was used as a control for immature genotype. Results for the differentiated hiPSCs for both conditions were normalized to undifferentiated hiPSCs (experimental negative control). Data presented as mean ± SD (n = 3). *p < 0.05; **p < 0.01; ***p < 0.001.

The results of q-PCR for both conditions, i.e. with and without HAMEC, demonstrated the presence of several gene markers in terminally differentiated HLCs (Fig. 3). However, hiPSC-EB + EC-HLCs showed a higher gene expression pattern for most of the markers when compared to hiPSC-EB-HLCs (Fig. 3). In particular Albumin (p < 0.01), HNF-4α (p < 0.01), HNF-1β (p < 0.0001), CYP1A2 (p < 0.0001), CYP2B6 (p < 0.0001) and UGT1A3 (p < 0.01) were statistically higher in hiPSC-EB + EC-HLCs when compared with hiPSC-EB-HLCs. Notably, for most of the genes tested, hiPSC-EB-HLCs and hiPSC-EB+ EC-HLCs showed a statistically higher gene expression when compared with human primary hepatocyte (HPH) and human neonatal hepatocyte (HNH).

**Figure 4:**
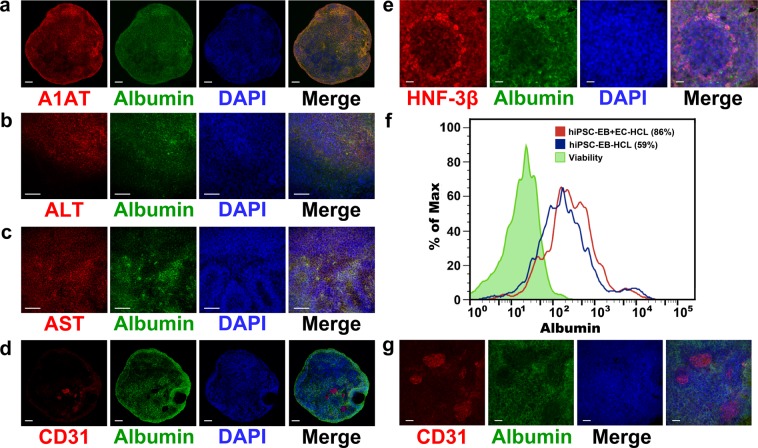
Tissue-specific marker analysis through immunofluorescence and FACS analysis. Following the differentiation program, terminally differentiated hiPSC-EB + EC-HLCs expressed mature hepatocyte-specific markers, as evidenced by the presence co-staining of (**a**) ALBUMIN and Alpha-1 Anti-Trypsin (A1AT), (**b**) ALBUMIN and ALT, (**c**) ALBUMIN and AST (**d**) ALBUMIN and CD31, (**e**) ALBUMIN and HNF-3β. Scale bar 100 µm. (**f**) FACS analysis for albumin positive cells showed a greater percentage of albumin positive cells in the condition with endothelial compared with the one without (86% vs 59%). (**g**) Magnified (60X) detail of ALBUMIN/CD31 positive cells showing the rosettes organization of CD31 positive cells interspersed with ALBUMIN positive cells. Scale bar 50 µm.

Furthermore, the immunofluorescence co-staining of terminally differentiated HLCs confirmed a robust expression of alpha-1 anti-trypsin (A1AT), alanine aminotransferase (ALT), aspartate aminotransferase (AST), CD31, and HNF-3β with albumin^42–44^ (Fig. 4a–e). Interestingly, CD31 positive cells displayed a rosette organization within the differentiated organoids, indicating that the HAMEC self-organized themselves in functional structures. Figure 4g shows a high magnification detail for the co-staining of CD31 and albumin at the end of the differentiation process. Differentiated HLCs without HAMEC did not show any CD31 positive cells (Supplementary Fig. 3 lower panel).

By FACS analysis, hiPSC-EB + EC-HLCs showed an increased amount of albumin positive cells when compared to the condition without HAMEC (86% vs 59% respectively) (Fig. 4f).

### hiPSC-EB + EC-HLCs displayed improved protein secretion *in vitro*

**Figure 5:**
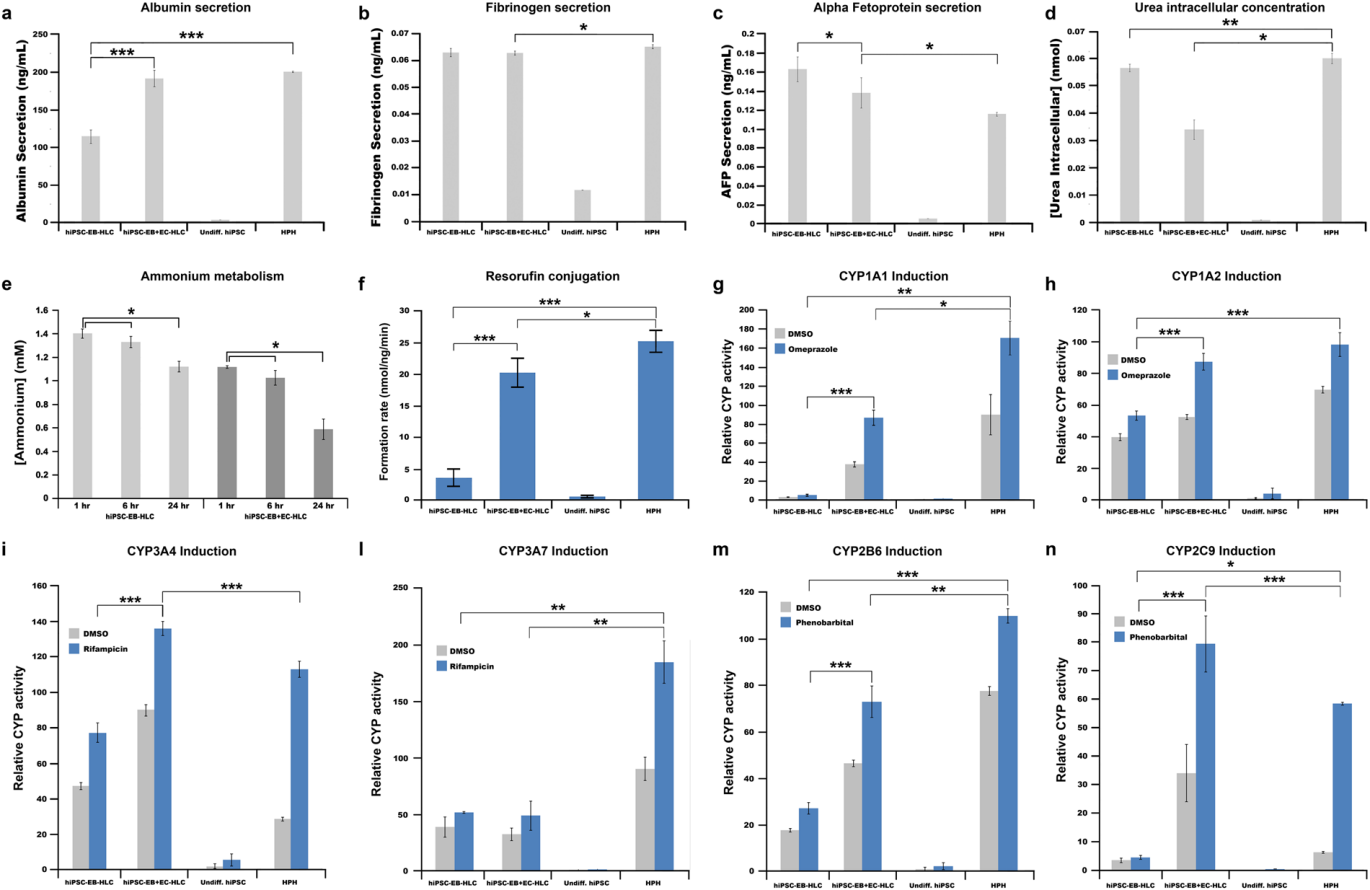
Secretion pattern of several hepatic proteins by hiPSC-EB-HLCs. Conditioned media from hiPSC-EB-HLCs were collected after 48 hours from the completion of the differentiation protocol for both conditions with and without endothelial cells. (**a**) Albumin, (**b**) fibrinogen and (**c**) Alpha Fetoprotein (AFP) were detected in the medium and (**d**) intracellular Urea was detected. Differences in secretion between the conditions with endothelial cells were statistically significant with respect to the condition without endothelial cells for the Albumin and AFP. There was not statistically significant difference between the two experimental conditions for the Fibrinogen and Urea intracellular concentration. Undifferentiated hiPSCs were used as negative control, and human primary hepatocyte as positive control. The results are representative of at least three independent experiments. Data presented as mean ± SD (n = 3). *p < 0.05; **p < 0.01; ***p < 0.001; Detoxification property analysis of the differentiated HLCs. (**e**) The ammonium metabolism assay conducted on a period over 24-hour for both conditions with and without endothelial cells showed a higher ability of ammonium clearance for the hiPSC-EB + EC-HLCs (about 45% from the first hour) when compared with hiPSC-EB-HLCs (about 20% from the first hour); (**f**) Phase II detoxification analysis through resorufin conjugation assay: The results showed a higher formation rate for the condition hiPSC-EB + EC-HLCs compared with the hiPSC-EB-HLCs, reaching similar level of the HPH used as positive control. (**g**–**n**) Cytochrome P450 (CYP450) induction analysis: Several CYP enzymes were assessed through incubation of the differentiated HLCs with specific inducers: Omeprazole for the (**g**) CYP1A1, and (**h**) CYP1A2; Rifampicin for the (**i**) CYP3A4, and (**l**) CYP3A7; and Phenobarbital for the (**m**) CYP2B6, and (**n**) CYP2C9 for a period of 72 hours. DMSO was used as control to test the basal activity of the different CYP450. Data presented as mean ± SD (n = 3). *p < 0.05; **p < 0.01; ***p < 0.001.

hiPSC-EB + EC-HLCs demonstrated increased albumin secretion when compared with hiPSC-EB-HLCs (190 ng/ml vs. 115 ng/ml for 5×10^5^ cells respectively; p < 0.01) (Fig. 5a). This represented 95% and 57% respectively of the *in vitro* albumin production by the plated HPH (190 ng/ml vs. 199 ng/ml, p = 0.2426; 115 ng/ml vs. 199 ng/ml, p < 0.01).

Fibrinogen secretion was similar between the two conditions (0.0664 ng/ml vs. 0.0665 ng/ml, p = 0.21), while AFP showed a reduced secretion in the condition hiPSC-EB + EC-HLCs in comparison with hiPSC-EB-HLCs (0.138 ng/ml vs. 0.163 ng/ml, p = 0.02) (Fig. 5b,c). Both AFP and fibrinogen in the conditions with HAMEC showed total protein concentration at levels that were similar to those of HPH (AFP: 0.138 ng/ml vs. 0.179 ng/ml, p = 0.01; fibrinogen: 0.0665 vs. 0.0667, p = 0.02).

Intracellular urea concentration in hiPSC-EB-HLCs with HAMEC was significantly lower than HPH, in contrast to the hiPSC only group which exhibited comparable results to the HPH (0.0339 nmol vs. 0.06 nmol, p = 0.0091; 0.0564 nmol vs. 0.06 nmol, p = 0.0174 respectively) (Fig. 5d). However, this disparity could be attributed to difference in cell seeding density ratio between the two conditions, as HAMEC form almost 1/3 of the entire EBs in the HAMEC group, thus considerably decreasing the absolute number of functional HLCs capable of urea generation in the HAMEC group. Undifferentiated hiPSCs were used as negative control, in which production of the proteins was absent at all times (p < 0.01) (Fig. 5a–d).

### Metabolic P450 enzyme function improved in HLCs with HAMEC

Ammonia metabolism was evaluated by addition of 1 mM ammonium chloride (NH_4_Cl). While there was a steady decrease in ammonium concentration in both conditions, hiPSC-EB + EC-HLCs demonstrated a larger decrease in ammonia compared to the cells without HAMEC (48.21 ± 2.61% vs. 20.34 ± 4.21% respectively; P < 0.01) (Fig. 5e).

Resorufin conjugation is a measure of phase II detoxification, and the assay measures the increase in fluorescence by resorufin, when the 7-ethoxy terminal is cleaved by CYP1A1/2 enzymes within cells^45,46^. We observed a concentration of resorufin in the hiPSC-EB + EC-HLCs condition that was closer to the HPH group (20.3 vs 25.34 nmol/ng/min), and higher compared to hiPSCs only (20.3 vs. 3.58 nmol/ng/min) (Fig. 5f).

Liver detoxification capacity of the HLCs *in vitro* was measured by characterizing the activities of six Cytochrome P450 (CYP450) enzymes (CYP1A1, CYP1A2, CYP3A4, CYP3A7, CYP2B6, and CYP2C9). DMSO was used as a control in cell culture to test the basal activity of the different CYP450. Our results indicate significant increases in the activities of all the tested isoforms of CYP450 relative to the DMSO control (Fig. 5g–n). Specifically, hiPSC-EB + EC-HLCs displayed increased CPY450 activity when compared to those without HAMEC in response to induction with Omeprazole (**CYP1A1**: 86.19 ± 8.16% vs. 4.87 ± 0.87%; **CYP1A2**: 87.33 ± 3.54% vs. 53.33 ± 4.13%), Rifampicin (**CYP3A4**: 135.67 ± 5.28% vs. 76.67 ± 4.45%; **CYP3A7**: 48.65 ± 12.89% vs. 51.49 ± 0.79%), and Phenobarbital (**CYP2B6**: 73.67 ± 3.18% vs. 27.33 ± 2.45%; **CYP2C9**: 79.86 ± 9.8% vs. 4.94 ± 0.70%).

Following induction, the hiPSC-EB + EC-HLCs displayed higher CYP450 activity relative to HPH for CYP3A4 and CYP2C9 isoforms (CYP3A4: 135.67 vs. 116.22, p < 0.01; CYP2C9: 79.86 vs. 3.43, p < 0.01), and lower CYP450 activities for CYP2B6, CYP3A7, and CYP1A1 isoforms compared to HPH (CYP2B6: 73.67 vs. 110.33, p < 0.01; CYP3A7: 48.65 vs. 235.79, p < 0.01; CYP1A1: 86.19 vs. 185.24, p = 0.02). In comparison, hiPSC-EB-HLCs had lower CYP450 activities for CYP1A1, CYP3A7, CYP1A2, CYP2B6, and higher CYP450 activity for CYP2C9 when compared with HPH. Undifferentiated hiPSC-EBs did not demonstrate any activities of the tested isoforms of CYP450 (Fig. 5g–n).

### Absorption and release of ICG, Glycogen storage and lipid processing was preserved in hiPSC-EB + EC-HLCs

**Figure 6:**
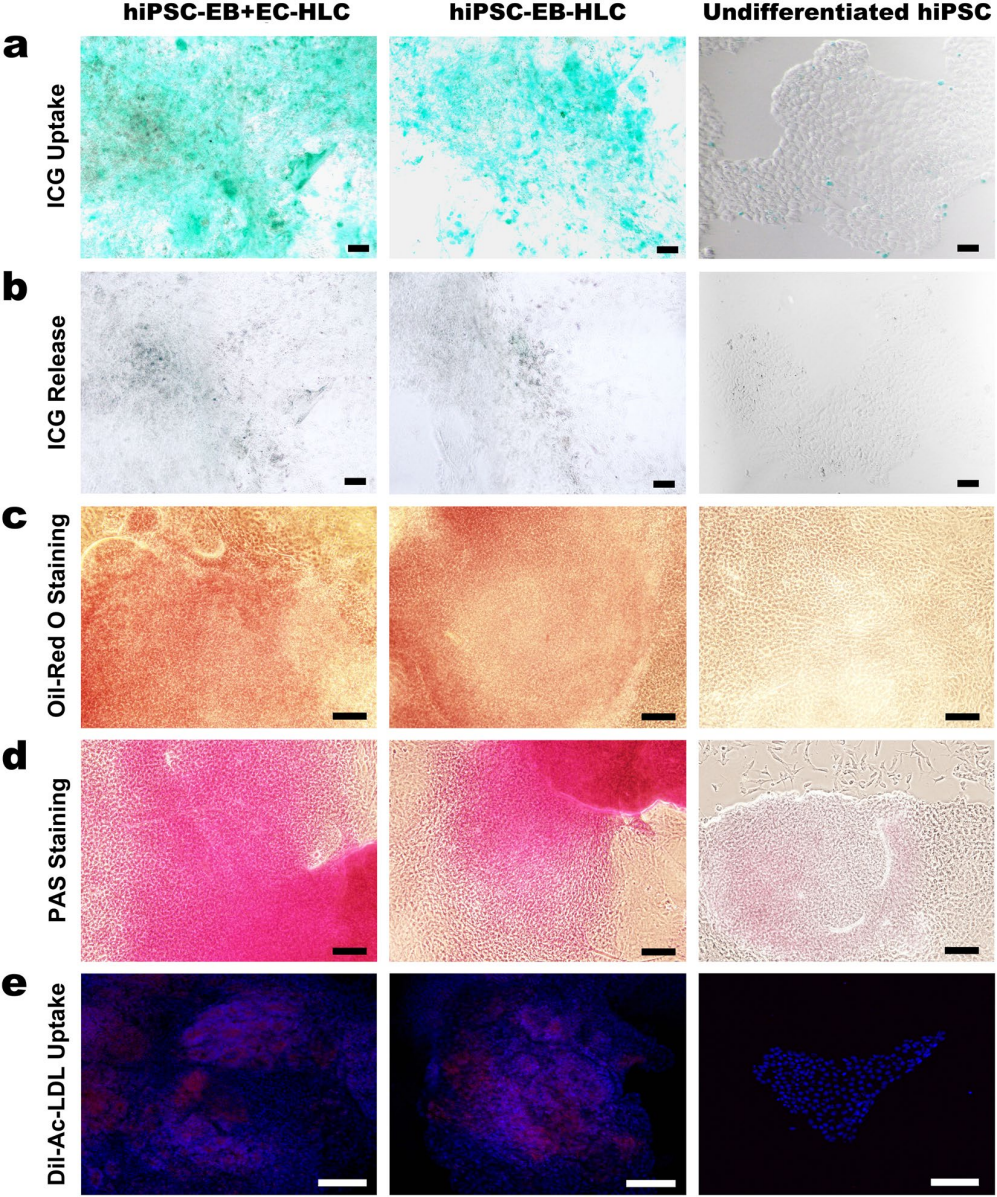
Ability of the differentiated HLCs to store different metabolites. The resultant hiPSC-EB-HLCs and hiPSC-EB + EC-HLCs showed hepatocyte functional activities, such as (**a**) Indocyanine green (ICG - Cardiogreen) uptake; (**b**) ICG release after 6 hours; (**c**) cytoplasmic accumulation of neutral triglycerides and lipids indicated by Oil-Red O staining; (**d**) glycogen storage indicated by PAS staining; and (**e**) Acetylated low-density lipoprotein (DiI-ac-LDL) uptake in red. Both conditions with and without endothelial cells showed a similar ability for all the metabolites assessed. Undifferentiated hiPSCs were used as negative control. Scale bar 100 μm.

We assessed the ability of differentiated HLCs for different metabolites storage. The results showed equivalent function to HPH with respect to indocyanine green (ICG - Cardiogreen) absorption and release after 6 hours (Fig. 6a,b), cytoplasmic accumulation of neutral triglycerides and lipids (Fig. 6c), glycogen storage (Fig. 6d), and acetylated low-density lipoprotein (DiI-ac-LDL) uptake (Fig. 6e)^47,48^. Undifferentiated hiPSCs were used as negative control (right column of Fig. 6) and did not show any of the functions studied.

### Stage-specific N- and O-glycan profiling of differentiated HLCs with and without HAMEC

**Figure 7:**
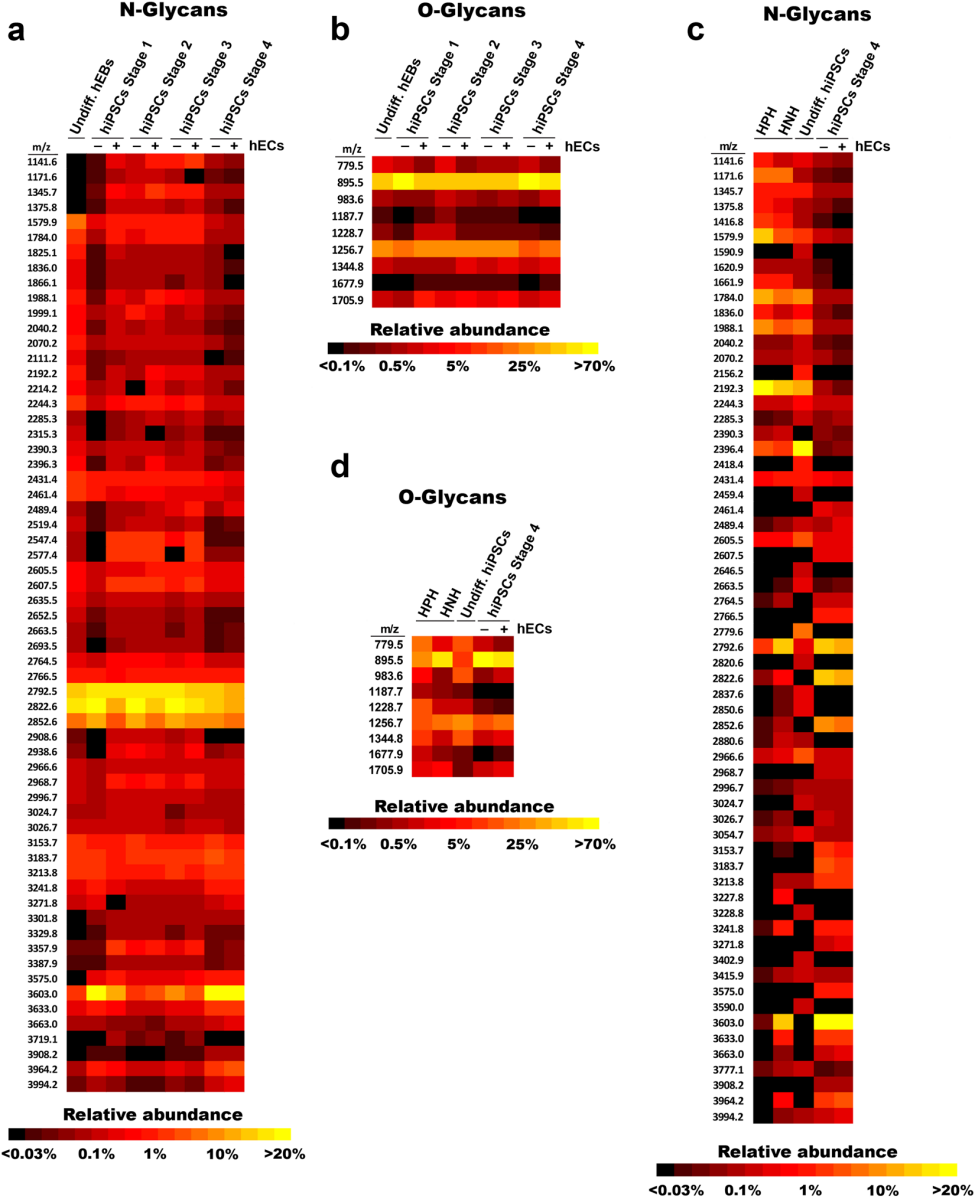
Mass spectrometry N- and O-glycan analyses of stage-specific hiPSCs differentiation. (**a**) Heat map representation of the relative abundance of the N-glycans identified in undifferentiated hEBs (Undiff. hEBs), hiPSCs Stage 1, hiPSCs Stage 2, and hiPSCs Stage 3, with (+) or without (−) co-culture of HAMEC. N-glycans with a relative abundance of less than 0.2% in all cell conditions are not represented here. (**b**) Heat map representation of the relative abundance of the O-glycans identified in Undiff. hEBs, hiPSCs Stage 1, hiPSCs Stage 2,and hiPSCs Stage 3, + or − co-culture of HAMEC. (**c**) Heat map representation of the relative abundance of the O-glycans identified HPH, HNH, Undiff. hiPSCs and hiPSC Stage 4 + or − co-culture HAMEC. (**d**). Heat map representation of the relative abundance of the N-glycans identified in human primary hepatocytes (HPH), human neonatal hepatocytes (HNH), undifferentiated hiPSCs (Undiff. hiPSCs) and hiPSC Stage 4 with (+) or without (−) co-culture HAMEC. N-glycans with a relative abundance of less than 0.2% in all cell conditions are not represented here.

Glycosylation is a major post-translational modification of cell-surface proteins modulating cell recognition, adhesion and signaling events^49–51^. Here we assessed by mass spectrometry (MALDI-ToF) the profiles and compositions of N- and O-glycans throughout the four HLCs differentiation stages. Although undifferentiated hEBSs and differentiated HLCs showed comparable N-glycan profiles, we found a much lower relative abundance of high-mannose-type N-glycans in HLCs compared to hEBs. A lower abundance of high-mannoses is a feature usually observed in tissues, as opposed to cells in culture, which commonly exhibit a high abundance high-mannoses. By contrast, the differentiated HLCs showed a relative higher abundance of complex sialylated N-glycans when compared to hEBs (Fig. 7a and Supplemental Fig. 4a). The O-glycan profile remained relatively unchanged throughout the differentiation process (Fig. 7b and Supplemental Fig. 4b).

We also analyzed the N- and O-glycan profiles of HPH, Human Neonatal Hepatocytes (HNH) and undifferentiated hiPSCs and compared them to the HLCs glycan profiles. The N-glycan profile of the HPH and undifferentiated hiPSCs were similar, with an overall relative high abundance of high-mannose-type N-glycans, constituting greater than 50% of the total N-glycans identified. The HNH N-glycan profile was similar to the HLCs as there was a lower proportion of high-mannose-type N-glycans and a relatively higher abundance of complex-type N-glycans (Fig. 7c and Supplemental Fig. 4a). However, the N- Glycan profile of our HLCs was similar to the one in fresh isolated HPH in observed previous studies^37^.

The O-glycan analyses also revealed a higher similarity between the HPH and undifferentiated hiPSCs than with the HNH and HLCs. While the mono- and di-sialylated core-1 O-glycans represented the vast majority of O-glycans in HNH (~90%) their abundance was not greater than 45% in HPH and undifferentiated hiPSCs (Fig. 7d and Supplemental Fig. 4b). These results demonstrate that the N- and O-glycan expressions and their variations are consistent with cellular differentiation.

### Transplantation of hiPSC-EB-EC-HLCs resulted in prolonged human albumin release *in vivo*

**Figure 8:**
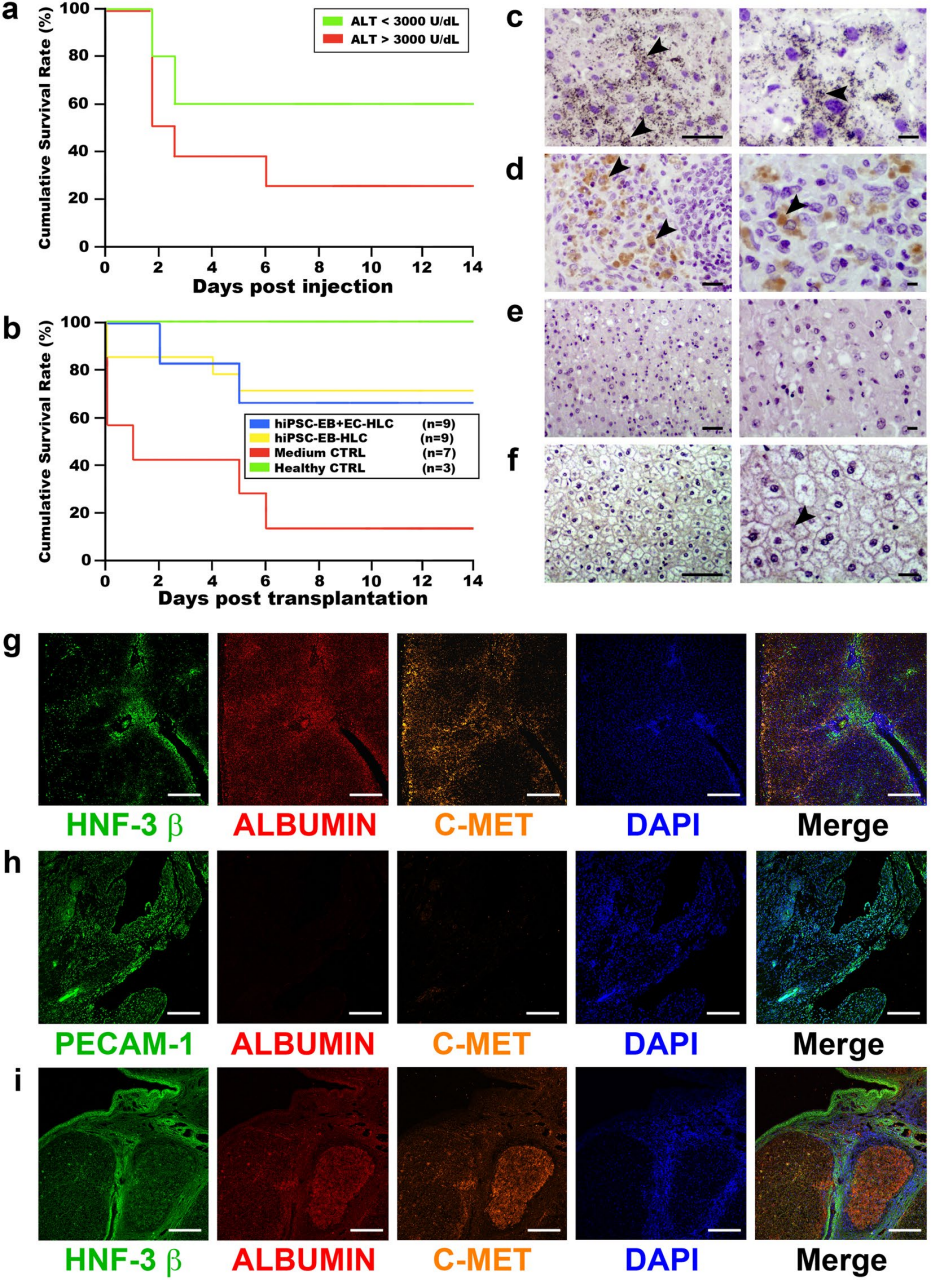
*In vivo* transplantation of hiPSC-EB-HLCs with and without HAMEC in a D-Galactosamine induced acute liver failure rat model. (**a**) Kaplan–Meier survival curve of 10- to 14-week-old control animals. 90% (8 of 9) of the rats that acquired liver injury with values of alanine aminotransferase (ALT) > 3,000 U/L at 1 day after injection died within 3-days, compared with a 3-day mortality (2 of 5) in those with an ALT < 3,000 U/L; (**b**) Kaplan–Meier survival plot of animals after HLCs transplantation with and without HAMEC, showed a similar survival rate between the two experimental conditions; (**c–f**) Representative liver and spleen sections with immunohistochemical staining. Background staining with hematoxylin-eosin. (**c**) Image of rat liver section transplanted with hiPSC-EB + EC-HLCs (20X left panel and 40X right panel). Double immunostaining was performed using a non-rat cross-reactive antibody to human albumin and non-rat cross-reactive antibody to human EC marker platelet EC adhesion molecule (PECAM)-1. Black arrows indicate presence of intracellular human albumin but there was not presence of PECAM-1. (**c**) Image of a rat spleen section transplanted with hiPSC-EB + EC-HLCs (20X left panel and 40X right panel). Double immunostaining was performed using a non-rat cross-reactive antibody to human albumin and non-rat cross-reactive antibody to human EC marker platelet EC adhesion molecule (PECAM)-1. Black arrows indicate human PECAM-1 positive ECs within the spleen but not the presence of human albumin. (**e**) Liver section from a control rat that was transplanted with medium only (negative control). Section was stained for human albumin and human PECAM-1 (10X on left panel and 20X on right panel). The sections show the absence of staining for both antibodies, indicating the specificity for human species of the antibodies used. (**f**) Normal human liver section (positive control) stained for human albumin (10X on left panel and 20X on right panel). Black arrows indicate presence of human albumin both intracellularly and extracellularly. Scale bar 2.5 µm. (**g**–**i**) Representative liver and spleen sections with immunofluorescence staining. (**g**) Representative image of liver section from a rat transplanted with hiPSC-EB + EC-HLCs. Immunofluorescence staining with non-rat cross-reactive antibodies to hepatocyte markers human HNF-3β, human albumin, and human C-MET displayed the presence of all 3 markers. (**h**) Representative image of a spleen section from a rat transplanted with hiPSC-EB + EC-HLCs. Immunofluorescence staining with non-rat cross reactive antibodies to PECAM-1, human albumin and human C-MET display presence of human PECAM-1 but no presence of human albumin or C-MET. (**i**) Normal human liver section (positive control) stained for human HNF-3b, human albumin and human C-MET. Image displays presences of all 3 markers. Nuclear staining with DAPI. Scale bar represents 100 µm.

D-galactosamine induced diffuse hepatic necrosis within 24 to 48 hours after administration in an immune-deficient rat model for acute liver failure, resulting in irreversible liver damage with 100% mortality within 6 days after induction (Fig. 8a and Supplementary Fig. 5)^33^. Transplantation of HLCs into the splenic capsule 14 to 16 hours after liver injury induction resulted in improvements in serum markers of liver injury (ALT). Mean ALT values increased after induction of liver failure (3652 U/L vs 58 U/L), and subsequently normalized 70 U/L following transplantation of hiPSC-EB + EC-HLCs and 294 U/L for the hiPSC-EB-HLCs (Data not shown). 66.7% of the animals transplanted with hiPSC-EB-HCLs and hiPSC-EB + EC-HCLs survived to 14 days after induction of liver failure, as compared to only 14% of negative control (Fig. 8b; Table 1).

**Table 1:** Transplantation of HLCs into ALF rat model.

	Survival to 14 days	Mean survival	Average human albumin in rat serum	Human albumin 3 days after transplant	Human albumin 14 days after transplant
hiPSC-EB-HLC only (n = 9)	66.7% (4/6)	11.0 ± 4.4	2.6 ± 0.24 ng/ml	50.0% (4/8)	0% (0/6)
hiPSC-EB-HLC + EC (n = 9)	66.7% (6/9)	11.2 ± 4.2	14 ± 2.08 ng/ml	50.0% (3/6)	50.0% (2/4)
Healthy Control (n = 3)	100% (3/3)	14.0	0	0% (0/3)	0% (0/3)
Medium Control (n = 7)	14.4% (1/7)	5.4 ± 4.5	0	0% (0/7)	0% (0/1)

During the 14 days post-transplantation observation period, there was a similar trend between the two conditions hiPSC-EB-HLCs and hiPSC-EB + EC-HLCs, and did not reach statistical significance (11.0 ± 4.4 vs. 11.2 ± 4.2 days respectively, p = 0.5802). An average of the 14 days post-transplantation human albumin that was detected in the serum of the survived animals receiving hiPSC-EB + EC-HLCs showed a greater amount (14 ± 2.08) when compared with the ones receiving the hiPSC-EB-HLCs (2.6 ± 0.24). During the 14 days post-transplantation, human albumin was detected in 50% of the rats at 3 days post-transplantation, while at 14 days post-transplantation only 50% of the rats transplanted with hiPSC-EB + EC-HLCs showed human albumin presence.

Notably, circulating human albumin in the rat serum was detected in both hiPSC-EB-HLCs with or without HAMEC at three days post transplantation. At 14 days, however, none of the hiPSC-EB-HLCs animals had detectable human albumin in the serum, while 50% of the hiPSC-EB + EC-HLCs of the animals had detectable circulating human albumin (Table 1).

After transplantation, double immunohistochemistry staining using non-cross-reactive human albumin and human-PECAM-1 was performed in all the transplanted rats to detect the presence of HLCs and HAMEC markers in their liver and spleen sections (Fig. 8c–e). Figure 6c–e show a representative set of images from sections of rats transplanted with hiPSC-EB + EC-HLCs or hiPSC-EB-HLCs, displaying the presence of human albumin in the liver but not in the spleen (Fig. 8c). In those rats transplanted with hiPSC-EB + EC-HLCs, human PECAM-1 for HAMEC detection was identified only in the rat spleens and not in the livers, indicating engraftment in the spleen (Fig. 8d). There was no human albumin or HAMEC staining in any sections among the controls (Fig. 8e). Human liver was used as positive control (Fig. 8f). Equally, immunofluorescence co-staining for the hepatic markers HNF-3 β, albumin, and C-MET in the rat liver sections showed distributed presence of human hepatocyte markers (Fig. 8g). The presence of human PECAM-1 in the spleen tissue was associated with the absence of human hepatocyte markers (Fig. 8h). Human liver was used as positive control in both immunostaining and immunofluorescence (Fig. 8f,i).

## Discussion

Current hepatic differentiation protocols giving rise to HLCs from hiPSCs are still limited by the low differentiation efficiency and high heterogeneity of the resultant cell populations^18,52–54^.

Our results show that the addition of HAMEC during hepatic differentiation of hiPSCs, using our already established hepatic differentiation protocol^21^, led to a consistently better performance in comparison to the hiPSCs only group across a wide spectrum of *in vitro* hepatic functions. These include liver-specific gene expression, cytochrome P450 activity, albumin, ammonia metabolism, and induction to a high level of a wide range of coagulation factors at both gene and protein level. *In vivo* performance of the transplanted HLCs improved albumin secretion. Using our hEBs formation technology, we were able to interlace HAMEC with hiPSCs in the same cluster, in a specific ratio of 1:3 respectively. This ratio was deemed optimal after several trials comparing different HAMEC:hiPSCs combination ratios based on differentiation yield in 3D culture (data not shown). The choice to use HAMEC was made in order to find a type of cell that would be always easily accessible, such as adipose tissue, averting the need for invasive procedures that might pose potential risks for the patients. To ensure that the presence of HAMEC interlaced with hiPSCs did not affect the progression of the differentiation process, adequate stepwise differentiation of our hiPSC-EB + EC-HLCs vs hiPSC-EB-HLCs was confirmed by stage-specific temporal protein expression and glycomic analyses, which resulted to be in line with previous landmark studies^55–60^.

Given the important role of ECs in embryogenesis, we sought to assess their potential impact on hepatic differentiation. Endothelial cells are one of the first cells to be present in the initial stages of embryogenesis^61,62^. Besides providing passive channels for distribution of blood and nutrients to various organs, ECs play an essential role in organogenesis, through creating vascular networks that secrete a wide range of organ-specific growth factors called “angiocrine factors”^63,64^. These factors are crucial elements that help orchestrate induction, specification, and guidance of organ generation processes, along with maintenance of homeostasis and metabolism. This signaling pathway is of utmost important during liver organogenesis, where ECs help induce liver bud generation prior to the circulation of body fluids within the developing organ^61^. Our main hypothesis was that HAMEC interlaced with hiPSCs would further help improve our clusters differentiation yield and phenotype in addition to post-transplantation performance through recruitment of host blood vessels, stimulation of endogenous factors release by the host endothelial cells in response to angiocrine signals sent by endothelial in the hiPSC-EB + EC-HLC clusters.

Co-culture of hiPSCs with supporting cells has been extensively investigated; influencing the specific co-culture of hiPSCs with HAMEC initiated in this study^55–60,65,66^. Tekebe *et al*. reported the formation of functional liver buds through co-culturing hiPSCs with human umbilical vein endothelial cells (HUVEC) and mesenchymal stem cells (MSCs). They observed that culturing hiPSCs-derived HLC with HUVEC alone did not lead to the formation of 3D hepatic buds. However, the resultant expression profiles of these co-cultured cells had more resemblance to human hepatic tissue than hiPSCs-HLC differentiated without HUVEC^67,68^ as we observed in our results when HAMEC were interlaced with hiPSCs. Another study examined the differentiation yield of hiPSCs and hECs co-cultured in a multicomponent of hydrogel fibers, showing that presence of hECs in the tissue construct improved the scaffold vascularization after implantation in a mouse with partial hepatectomy^56^. Similarly, improved hepatic functions have been reported using 3D bio-printing of *in vivo*-like hepatic lobules. The tri-culture model composed of hiPSC with HUVEC and adipose-derived stem cells had superior performance, in comparison to the 2D monolayer and the 3D-hiHPCs only model^69^. All these findings are consistent with our study, where the addition of HAMEC to the hiPSCs resulted in significant improvement across all tested human hepatic functions. Recently, Freyer *et al*. reported divergent data, showing that addition of HUVEC did not improve the differentiation outcome of hiPSCs. Authors concluded that culture media composition had more important effect on differentiation outcome than presence of HUVEC^70^. These findings may in fact support the importance of a specific ratio between the amount of hiPSCs and hECs, as well as the cell to cell interactions of our 3D hEBs technology. At the end of the differentiation process we wanted to study the distribution and number of HAMEC in comparison with the initial cell seeding ratio. We observed that HAMEC self-organized themselves in rosettes structures within the organoids, and their ratio changed from the initial 33% to about 15%.

Assessment of CYP activity represents an important parameter to test liver phase I detoxification enzymes. In addition to baseline constitutive activity, the hiPSC-EB-HLCs from both groups (with and without HAMEC) demonstrated the ability to up-regulate specific P450 family CYP enzyme isoforms in response to the appropriate inducers. Six major and physiologically important P450 isoforms were tested in our HLCs, including both adult and foetal enzymes^71,72^. In all of the P450 enzymes analyzed the hiPSC-EB + EC-HLCs showed greater induction ability when compared with the hiPSC-EB-HLCs. Furthermore, liver phase II detoxification abilities were assessed via the resorufin conjugation assay, which showed a higher formation rate in the clusters with HAMEC when compared to the hiPSC only group, and a similar value compared to the HPH. These results suggest that the clusters hiPSC-EB + EC-HLCs have achieved a better hepatic maturation and are functional in regards to detoxification and metabolism when compared with the hiPSC-EB-HLCs^73^.

Coagulation capabilities of our HLCs were studied by q-PCR and protein secretion analysis. Production of coagulation factors by differentiated hiPSCs have not been fully characterized in the literature. To the best of our knowledge, this is the first study reporting a comprehensive gene and protein secretion analysis of hiPSC-derived HLCs. Almost all the previous works published on coagulation factors have used differentiated animal-derived pluripotent stem cells^38,39,41^. HLCs generated from hiPSCs derived from Haemophilia A patients’ urine sample (HA-iPSCs), was reported by Jia *et al*. In this study they showed that HA-iPSCs derived HLCs could recapitulate the phenotype of HA *in vitro*, having a functional HLCs which failed to produce FVIII^74^. Interestingly, our hiPSC-EB + EC-HLCs showed a higher level of FVIII gene expression and protein secretion, when compared to either hiPSC only or HPH, providing a promising modality for these types of diseases. q-PCR for both experimental groups, showed adequate gene expression levels of most factors involved in both the coagulation and subsequent thrombolysis pathways, with hiPSC-EB + EC-HLCs group demonstrating statistically significant higher levels than the HPH, HNH and hiPSC-EB-HLCs groups, for the majority of factors.

Gene expression level is not always correlated with protein secretion, as this last one is often correlated to the level of cell maturation and to the stimuli applied to the cells in culture^75–78^. Quantitative secretion of various coagulation factors was performed after incubating our organoids for 4 hours with TNF-4 α, which is well-known to induce coagulation factors activation^79,80^, with subsequent comparison of the two experimental groups. Data showed a general trend for HPH starting at a higher level of coagulation factors secretion compared to both groups. Over time, the well-documented phenomenon of HPH losing their function at 7–10 days in culture resulted^38,39,41,73,81–84^. Our experimental conditions showed comparable or higher level of coagulation factors secretion at all later time points in the hiPSC-EB + EC-HLC. We then compared coagulation factors normally produced by hECs (i.e. factors VIII and vWF) between the two experimental groups, and we observed that hiPSC-EB-HLCs did not secrete any of these factors at any time, while hiPSC-EB + EC-HLCs had an increased secretion over the one-week period study. These data suggests that our 3D model supports integration of the HAMEC to express functional proteins that could be useful for potential therapy in haemostatic related diseases.

Factor X has a pivotal role in the coagulation cascade. Therefore, we sought to test the physiological capacity of our HLCs for the factor Xa (FXa) generation^85^. Our data showed that hiPSC-EB-HLCs and hiPSC-EB + EC-HLCs, were able to generate FXa. Importantly, there were no significant differences in the Vmax between the two conditions; however the presence of HAMEC in the clusters increased the affinity of the FXa generating enzyme, as evidenced by the marked decreased Km in comparison to the hiPSCs only. This is a further indication of a functional integration of the HAMEC within our HLCs. The ability of our hiPSC-EB + EC-HLCs to generate FXa makes them a viable target for potential therapeutic intervention in pathologically altered blood coagulation. Together with FXa generation, another important element in the coagulation cascade is the thrombin generation, which allow for the formation of clot during vessel damage. Our differentiated HLCs demonstrated the ability to generate thrombin in an *in vitro* assay, showing a statistically greater ability to generate thrombin even in comparison with HAMEC and HPH that were used as positive controls.

Glycosylation is a major modification of cell-surface lipids and proteins that plays a role in numerous biological processes including cell signaling^49^, cell-recognition and adhesion events^50,51^. The importance of cell-surface glycan has been demonstrated in the context of stem cell biology as several glycan epitopes have already been identified^86–88^. Here, we have analyzed the N- and O-glycosylation changes occurring during hiPSC differentiation into HLCs. In concordance with prior studies, we observed a relatively high abundance of high-mannose type N-glycans in undifferentiated hiPSCs and undifferentiated hEBs. By contrast, our stage 3- and stage 4-differentiated hiPSCs showed a dramatic reduction of the relative abundance of high-mannose type N-glycans associated with an increase in relative abundance of complex N-glycans. Interestingly, the N-glycan profile of terminally differentiated HLCs is very similar to the N-glycan profile of the freshly isolated HPH, as reported by Montacir *et al*.^37^. From this perspective, the difference in our new differentiation protocol clearly led to an N-glycan profile more mirrored to HPH. Cryo-preserved HPH used in this study showed a different N-glycan profile that was enriched in high-mannose-type glycans and with a relatively lower abundance in complex-type N-glycans, which is typical of cells in adherence culture. On the other hand, the N-glycan profile demonstrated by our HLCs was similar to the native tissue glycan profile, suggesting that the 3D technology used to generate our HLCs led to a self-organization of the cells that resemble that observed in organs. As far as we know, our analyses of the O-glycans of hiPSCs differentiating into HLCs are the first of their kind in this type of differentiation protocol. Unlike the N-glycan profiles, the O-glycan profiles only showed minor variations between the different samples we analyzed as previously reported^89^.

Using a D-Galactosamine acute liver failure rat model, we compared *in vivo* performance between both HLCs groups. Experiments were carried out using HLCs with and without interlaced HAMEC. HLCs were injected within the splenic capsule, and animals were observed for two weeks. By the end of the observation period, numerous HLCs were seen in the recipient rat livers, with no HLCs identified in the spleen, a finding consistent with prior reports^7,90^. These transplanted cells were able to bridge the animals through the critical period of acute liver failure, showing persistent secretion of human albumin in the rat serum throughout the observation period. In particular, animals transplanted with hiPSC-EB + EC-HLCs displayed a more sustained and higher concentration of human albumin in their serum compared to the ones transplanted with hiPSC-EB-HLCs. The comparison between the two conditions showed that addition of HAMEC to hEBs improved the differentiation process as evidenced by sustained hepatic function of the resultant hiPSC-EB + EC-HLCs. Interestingly, in contrast to HLCs, HAMEC remained in the spleen without migrating to the liver, suggesting that the role of HAMEC may be in the initial stages of engraftment and nidation of the HLCs.

Here, we report a strategy to improve hepatic differentiation yield, with a general amelioration of all the hepatic *in vitro* and *in vivo* performances of differentiated hiPSCs through the co-culture with HAMEC. This technique could represent a new potential technique that could be used for cell transplant therapy.

## Materials and Methods

### Cell sources and culture conditions

Human induced pluripotent stem cells (iPSCs) were a foreskin fibroblast-derived cell line iPS(foreskin)-3 (purchased from WiCell Research Institute, Madison, WI – cat# WB0002) and cultured in chemically defined stem cell medium (mTeSR1 basal medium with mTeSR1 supplement, Stem Cell Technologies, Ontario, Canada) on a Matrigel matrix (BD Biosciences, San Jose, CA). iPSC colonies were passaged using Versene (EDTA) (Lonza, Allendale, NJ) for 8 minutes at room temperature.

Human Adipose Microvascular Endothelial Cells (HAMEC) were purchased from ScienCell Research Laboratories (ScienCell Research Laboratories, Carlsbad, CA – cat# 7200) and cultured in Endothelial Cell Medium (ScienCell Research Laboratories - ECM, Cat# 1001) on fibronectin coated flasks (2 µg/cm^2^) (ScienCell Research Laboratories - Cat# 8248) according to the manufacturer protocol.

Human hepatocytes were isolated from a liver wedge (LifeNet/UNOS), using the three step perfusion technique (collagenase-CiZyme), within a laminar flow hood that contained a circulating 37 °C water bath with a sterile containment bowel bag. After perfusion, the liver capsule’s catheters and tubing were removed from its sterile bag and the capsule was gently placed into a sterile beaker. The isolated hepatocyte cells were released into the sterile beaker (containing chilled EMEM (Charles River) media), using sterile scissors to cut into the softened liver matrix. This cell mixture was filtered using proper sterile mesh over a sterile container and then the filtered cell mixture was centrifuged at 4 °C at 400 rpm. All liver cell pellets were washed three times in HBSS (Charles River) and stored in a two liter sterile bottle containing fresh EMEM media, on wet ice. After trypan blue exclusion assay, the hepatocytes were then centrifuged again at 450 rpm and cryopreserved in Belzers Solution (Viaspan) + 10% DMSO (Sigma) and crypreserved at −20 °C for 1–2 hours, then moved to the −80 °C freezer and the LN2 tank, until further use. Trypan Blue Exclusion Assay results ranged from 35% to 95% viable cells totalling 100 × 10^6^ to 10 × 10^10^ hepatocytes, depending on the variables of the case. For experimental procedures, HPH were thawed and initially cultured in collagen type I coated plates with Hepatocyte Thaw Medium (Thermo Fisher Scientific, Boston MA), and Primary Hepatocyte Thawing and Plating Supplements (Thermo Fisher Scientific) according to the manufacturer instructions. After overnight cell culture, HPH were cultured in Cryopreserved Hepatocyte Recovery Medium (CHRM – Thermo Fisher Scientific), and Hepatocyte Maintenance Supplement Pack (Thermo Fisher Scientific), according to the manufacturer instructions. HPH were used as positive control in all the experiments and were cultured for a period not longer than one week.

### Embryoid body (EB) formation

Using locally developed Teflon stamps and low melting point agarose (Sigma-Aldrich), agarose micro-well arrays were made^30,31^. The agarose, 40 g L^−1^, was then dissolved in phosphate buffered saline (PBS) at 100 °C and pipetted into the culture ware. The Teflon stamps were pressed into the agarose solution for approximately 5 minutes. The agarose gelled in about 2 minutes and the stamp was withdrawn with resultant micro-well arrays in the agarose gel substrate. Following the agarose gelling, we primed the arrays through incubation with EB differentiation medium (1:1 mixture IMDM and F-12 Nutrient Mixture (Ham) (Invitrogen), 5% foetal bovine serum (Invitrogen), 1% (vol/vol) insulin transferrin selenium-A supplement (Invitrogen), 55 μM monothioglycerol (Sigma-Aldrich), 100 U L^−1^ penicillin, and 0.1 mg L^−1^ streptomycin (Invitrogen) overnight at 37 °C and 5% CO_2_.

Then, 1.2 × 10^6^ dissociated hiPSC in a 50 μl suspension were placed in each micro-well array and allowed to sediment into the microwells for hiPSC-EBs formation. Three-dimensional EB were aspirated from the micro-wells and transferred to a 35 mm tissue culture dish (BD Biosciences) after 24-hour incubation at 37 °C. The cells were kept in suspension culture in basal hepatocyte medium under gentle agitation on an orbital shaker at 37 °C and 5% CO_2_ with medium changes every other day. For the formation of hEBs with HAMEC (hiPSC-EB + EC-HLC), hiPSCs and HAMEC were digested into a single cells suspension, and counted to precisely determine the right number to interlace them in a ratio of 3:1 respectively. The mixed hiPSCs and HAMEC where then plated in our hEBs agarose micromold technology for the cluster formation (Supplementary Figs 6a and 7)^31^.

### Hepatic differentiation procedure

Our four-stage *in vitro* hepatic differentiation protocol sought to recapitulate the *in vivo* changes occurring during embryogenesis^21^ (Supplementary Fig. 6b). The four stages are definitive endoderm, foregut endoderm, hepatobiliary progenitor and committed hepatocyte. With two every-other-day medium changes and addition of the soluble differentiation factors, each stage of the differentiation protocol lasted four days.

Basal differentiation medium consisted of IMDM with F-12 Nutrient Mixture (Ham), 5% foetal bovine serum, 1% (vol/vol) insulin transferrin selenium-A supplement, 55 μM monothioglycerol, 100 U ml^−1^ penicillin, and 0.1 mg ml^−1^ streptomycin (Sigma-Aldrich). The differentiation towards the definitive endodermal stage was promoted by addition of 10 ng ml^−1^ basic FGF, 100 ng ml^−1^ Activin-A and 10 ng ml^−1^ TGF-β (all from PeproTech, Rocky Hill, NJ). The second foregut endoderm stage was promoted through the addition of 10 ng ml^−1^ FGF-4 (PeproTech) and 10 ng ml^−1^ BMP-4 (Invitrogen). Following endodermal commitment, absence of Wnt signalling is integral to hepatobiliary differentiation. Suppression of Wnt signalling and promotion of the third stage of differentiation was done by addition of Wnt pathway inhibitors, 1 μg ml^−1^ WIF-1 (R&D System, Minneapolis, MN) and 0.1 μg ml^−1^ DKK-1 (PeproTech). Following hepatobiliary commitment, the presence of HGF and oncostatin M determines differentiation into cholangiocytes or hepatocytes. Through adding 50 ng ml^−1^ HGF (PeproTech) and 30 ng ml^−1^ Oncostatin M (PeproTech), we directed the hepatobiliary cells into a hepatocyte pathway at the fourth stage.

All factors were added to the cell culture media and the embryoid bodies were maintained in suspension through gentle orbital agitation for the embryoid body differentiation. Undifferentiated hEBs were collected from the same batch to be used as negative control before the application of the differentiation protocol. We conducted all the experiments for the hepatic differentiation with and without HAMEC were performed starting from the same batch of hiPSC-EBs; therefore, in order to ensure the reproducibility of our results, the samples were analysed all at the same time at the end of the differentiation process. All of the experiments were conducted in parallel, independently and in triplicate starting from different batches of hiPSCs with and without HAMEC, as well as the undifferentiated hiPSC-EBs (negative control), and primary adult human hepatocyte (positive control) to ensure the reproducibility of our results.

### HLCs morphology assessment

After terminal differentiation, HLCs were allowed to adhere into matrigel coated plates to form a monolayer. After monolayer formation, the morphology of the differentiated HLCs was evaluated by light microscopy at different magnifications.

### hEB viability

Cell viability was evaluated at the end of the differentiation process by LIVE/DEAD staining (Catalog # L-7013, Molecular Probes) to determine the presence of any core necrosis according to the manufacturer’s instruction. Fluorescent images were acquired with confocal microscopy using Olympus IX81.

### Immunofluorescence assay

At the end of each stage, embryoid bodies undergoing differentiation were collected for immunofluorescence analysis of stage-specific markers. After fixing the embryoid bodies with 4% (wt/vol) paraformaldehyde for 90 minutes, they were permeabilized with 0.3% (vol/vol) Triton-X 100 in PBS for 1 hour, and blocked with 0.5% (vol/vol) goat serum (Sigma-Aldrich) in PBS for 1 hour. Then, samples were incubated with the primary antibody at 4 °C for three days. Following several washes, samples were then incubated with the secondary antibody at room temperature for 2 hours. Probably due to the large radius of the EB clusters and increased time for diffusion, the above incubation times were necessary to ensure complete staining.

We used the following human specific primary antibodies: rabbit anti SOX17 (Santa Cruz, sc-20099; 1:100); mouse anti FOXA2 (Abcam, ab60721); 5 µg ml^−1^, goat anti Hhex (Santa Cruz, sc-15128; 1:100); mouse anti GATA-4 (Santa Cruz, sc-25310; 1:100); mouse anti AFP (Santa Cruz, sc-166325; 1:100); mouse anti HNF-4α (Santa Cruz, sc-8987; 1:100); goat anti Albumin (Santa Cruz, Santa Cruz, CA, sc-46293; 1:100); mouse anti Cytokeratin 18 (CK-18) (Abcam, ab82254, 5 µg ml^−1^); mouse anti HNF1-α (Santa Cruz, sc-135939; 1:100); rabbit anti human C-MET (Santa Cruz, sc-10; 1:100), mouse anti human AGXT2L2 (ALT) (Santa Cruz, sc-365670; 1:100), mouse anti human AATM (E-7) (AST) (Santa Cruz, sc-271702; 1:100), mouse anti human AAT (H-7) (A1AT) (Santa Cruz, sc-166018; 1:100), mouse anti HNF-3β (RY-7) (Santa Cruz, sc-101060; 1:100), and Alexa Fluor 488 mouse anti-CD31 antibody (Abcam; ab215911). The following secondary antibodies were used: Cy2-AffiniPure goat to mouse IgG; Fc Subclass 1 Specific (Jackson ImmunoResearch, 1:100); Cy2-AffiniPure Goat Anti-Mouse IgG, Fcγ Subclass 2a Specific (Jackson ImmunoResearch, 1:100); Cy3-AffiniPure Goat Anti-Mouse IgG, Fcγ Subclass 2b Specific (Jackson ImmunoResearch, 1:100); Cy3-AffiniPure goat to rabbit IgG (H + L) (Jackson ImmunoResearch, 1:100); Cy™3 AffiniPure Donkey Anti-Goat IgG (H + L) (Jackson ImmunoResearch, 1:100) and Cy5-conjugated AffiniPure rabbit to goat IgG (Jackson ImmunoResearch, 1:100). We then counter-stained the nuclei with 4'6-diamidino-2-phenylindole (DAPI) in PBS for 1 hour. Fluorescent images were acquired with confocal microscopy using Olympus IX81. The ratio between differentiated HLCs and HAMEC at the end of the differentiation protocol was established by counting the number of CD31 and albumin-positive cells over the total number of nuclei in each Z-stack-cross-section of our organoids, obtained with a confocal microscope Olympus FV3000, and averaged on a minimum of 20 random microscopic fields for each cluster by using FluoView 3000. Over 50 organoids were used for the ratio determination.

### Gene expression assay

Quantitative-PCR (q-PCR) was performed to verify the presence of characteristic gene markers of differentiation. Extracted RNA was treated with RNase-free DNase (Promega) and reverse-transcribed using an iScript cDNA synthesis kit (Bio-Rad) according to manufacturer instructions. Custom PrimePCR plates (Bio-Rad, 96 well, SYBR plate with 9 unique assays, Catalogue #10025217) with lyophilized primers of interest were used with SsoAdvanced Universal SYBR green and run according to the manufacturer instructions. The following amplification conditions were used for a total of 40 cycles: activation for 2 minutes at 95 °C, denaturation for 5 seconds at 95 °C, annealing at 60 °C for 30-second melt curve at 65–95 °C (0.5 °C increments) for 5 sec/step. CFX96 Touch (Bio-Rad) was used for the amplification and data was processed using CFX Manager 3.1 (Bio-Rad). The following genes for the hepatic function were evaluated: Albumin, Alpha-1-antitrypsin (A1AT), CK18, Ornithine transcarbamylase (OTC), Phenylalanine hydroxylase (PAH), HNF-1α, HNF-4α, HNF-1β, HNF-6, seven P450 isoforms (CYP1A1, CYP1A2, CYP1B1, CYP2B6, CYP2C9, CYP3A4, CYP3A7), UDP-glucuronosyltransferase 1 family, A1 (UGT1A1), UDP-glucuronosyltransferase 1 family, A3 (UGT1A3), UDP-glucuronosyltransferase 1 family, A4 (UGT1A4) and Alpha-fetoprotein (AFP). The following genes for the coagulation factors were evaluated: Factor VII, Factor XII, Factor IX, Factor VIII, Factor X, Factor V, Thrombin, Fibrinogen, Factor XIIIb, Protein C, Protein S, Antithrombin III, Plasminogen, ADAMSTS13 and VWF. GAPDH was used as the reference housekeeping gene. Values were normalized to the negative control (undifferentiated hiPSCs) and reported relative to the glyceraldehyde-3-phosphate (GAPDH) housekeeping gene. Error bars represent the standard deviation of three independent experiments. Data is presented as mean ± SD.

### Coagulation factors quantification assay

For the quantitation of the coagulation factors secreted by the cells in the medium, conditioned medium was collected every other day for 6 days, and the coagulation factors were quantified using the following Procarta-Plex assays from Thermo-Fisher Scientific according to the manufacturer protocols: Human Coagulation Panel 1 (6plex) Multiplex (EPX060-10824 RUO); Human Coagulation Panel 2 (3plex) Multiplex (EPX030-10823 RUO); Human Coagulation Panel 3 (4plex) Multiplex (EPX040-10825 RUO); Human Factor X Simplex Multiplex (EPX01A-12152 RUO) and Human Fibrinogen Simplex Multiplex (EPX01A-12153 RUO). For the reading of the samples we used a Magpix Luminex XMAP Technology and analysed the data with xPONENT 4.2 software from Luminex. As calibrator we used Reference Coagulation Control Plasma. Since we quantitate relatively to this reference plasma, our results are in %ref plasma. This reference plasma is well characterized and contains the single coagulation factors in a given percentage of NIBSC standards. The following coagulation factors were considered for each panel: Antithrombin, Factor V, Factor VII, Factor VIII, Factor X, Factor XIII Prothrombin, Protein C, Protein S, VWF. Results were normalized to the number of cells (1 million cells).

### Factor Xa generation assay

Prior starting the assay, hiPSC-EB + EC-HLCs, hiPSC-EB-HLCs, HPH and HAMEC and 3D HAMEC clusters were separately incubated for 4 hours with TNF-α (10 ng/mL) into a 96-well plate, and then washed 1 time with HBS-BSA (20 mM HEPES, 150 mM NaCl, 5 mM KCl, 2 mM CaCl_2_, and 1 mg/ml BSA). Immediately after washing the HLCs, 10 µL of Factor X (final concentration 100 nM) and 10 µL Factor Xa chromogenic substrate (final concentration 200 µM) were added in a final volume of 90 µL with HBS-BSA in each well containing hiPSC-EB + EC-HLCs, hiPSC-EB-HLCs, HPH, HAMEC and 3D HAMEC clusters in triplicate. Another 10 µL of Factor VIIa (final concentration 0.1 nM) were added to the previous solution to initiate the reaction. The plate was immediately placed in the SpectraMaxi3X and read at 405 nm (kinetic-UV/ABS) for PNA release detection over a period time of about 6 hours. Data obtained were analysed using Graph Pad Prism 5 and using a nonlinear regression XY analysis by a Michaelis-Menten equation. Mean, SD, N were used as error function.

### Thrombin generation assay

Conditioned media was collected after two days from terminal differentiation of our organoids and centrifuged at 3000 × g for 10 minutes to rid it of cellular debris. We collected the supernatant and centrifuged at 16000 × g for 20 min. The supernatant was removed and the isolated micro-particles (MP) were reconstituted in 100 μL of platelet free plasma (PFP). Aliquots of MP samples were added to a 96-well plate. Fluorescent thrombin substrate (3.0 mg mL-1 of Bz-Phe-Val-Arg-AMC, 4003131; Bachem Bioscience) was added in the presence of calcium (~10 mM) to initiate thrombin generation. The assay was run for 30 min in a Synergy™ HTX Multi-Mode Microplate Reader and analysed with Gen5 microplate reader and imager software. Data were normalized to the control HAMEC.

### FACS analysis and cell sorting

100 hiPSC-EB-HLC with and without HAMEC were digested using trypsin for 15 minutes at 37 °C after completion of the differentiation protocol. We utilized live/Dead Zombie NIR™ Fixable Viability Kit (719 nm 746 nm 100 – cat. 423105/423106) to assess viability. Albumin staining was performed intracellularly using the Fixation/Permeabilization Staining Buffer Set (eBioscience, San Diego, CA). We used the following monoclonal antibody for the FACS analysis: anti-Human Serum Albumin APC-conjugated Antibody (R&D SYSTEMS), and PE mouse anti-human CD31 (Biolegend, 303105). Data acquisition was performed on BD FACS Aria II instrument. Purity after sorting was routinely > 95%. The threshold was drawn based on the control sample stained for viability (live/dead stain) but not for albumin. We then drew a gate so that the frequency of this control sample was considered the zero. Then we considered all the events above the threshold in the stained samples to be positive, with the analysis performed using FlowJo software. Then the mean fluorescence intensities (MFIs) were calculated using the geometric mean of the appropriate fluorescence channel in FlowJo. Finally, Expansion Indices were determined using the embedded FlowJo algorithm.

### Albumin, Fibrinogen and AFP secretion assays

Conditioned medium coming from fully differentiated hEBs was collected and stored at −80 °C after 48 hours of the last change of medium. We then quantified albumin secreted from the differentiated embryoid bodies into the culture media using a Human Albumin ELISA kit (Abcam ab108788) following the manufacturer’s instructions. Alpha-fetoprotein secretion quantification assay was performed using an Alpha Fetoprotein Human SimpleStep ELISA kit (Abcam ab193765) according the manufacturer’s instructions. The quantification of Fibrinogen secretion into the culture supernatant was done using a Fibrinogen Human SimpleStep ELISA Kit (abcam – ab171578) following the manufacturer’s instructions. All the samples were carried out in triplicate.

### Intracellular urea content assay

Using the whole clusters that were digested with a specific buffer coming from a commercial Urea Assay Kit (abcam – ab83362), total Urea content within the differentiated hEBs was measured, according to the manufacturer’s instructions.

### Ammonia metabolism assay

After addition of ammonium chloride, we evaluated ammonia metabolism through changes in ammonia concentration in the cell culture supernatant over a 24-hour period. The standard 1 mM of NH_4_Cl was added to the culture dishes containing 100 differentiated embryoid bodies in suspension. We then collected supernatant, and using a colorimetric ammonia assay kit (BioVision, Milpitas, CA), ammonium concentration was measured at 1-, 6- and 24-hour intervals after NH_4_Cl addition.

### Resorufin conjugation assay

For the Resorufin conjugation assay, hiPSC-EB + EC-HLCs and hiPSC-EB-HLCs were incubated for 72 hours with rifampicin. The clusters were then transferred into an uncoated black 96-well plate in triplicate and re-suspended in a 50:50 HBSS/Resorufin solution (40 µL HBSS and 40 µL Resorufin sodium salt - Sigma-Aldrich) and then incubated in the dark at 37 °C for 30 minutes. At the completion of the incubation time all the samples were analysed at the same time and read in triplicate. The fluorescence was measured for each sample on a luminometer/spectrometer (SpectraMax i3x – Molecular Devices) at an excitation wavelength of 535 nm and an emission wavelength of 581 nm, with a sensitivity of 35 nm. Blank medium (HBSS) was also read in triplicate to be further subtracted as basal level of fluorescent. Quant-iT™ PicoGreen® dsDNA kit was used as fluorescent nucleic acid stain for quantitating double-stranded DNA (dsDNA) in solution. Using the PicoGreen® dsDNA quantitation assay, every well activity was normalized to total number of viable cells (double strand DNA).

### CYP activity assay

According to the manufacturer’s instructions, the Cytochrome P450 enzymes activity was performed using the P450-GloTM Assay Kit (Promega, Madison, WI). Different P450 enzymes activity were tested, in particular the CYP1A1 (P450-Glo CYP1A1 – V8752 – Promega, Madison, WI), CYP1A2 (P450-Glo CYP1A2 Induction/Inhibition – V8422 – Promega, Madison, WI), CYP3A4 (P450-Glo CYP3A4 (Luciferin-IPA) – V9002 – Promega, Madison, WI), CYP3A7 (P450-Glo CYP3A4/7 – V8902 – Promega, Madison, WI), CYP2B6 (P450-Glo CYP2B6 – V8322 – Promega, Madison, WI), and the CYP2C9 (P450-Glo CYP2C9 – V8792 – Promega, Madison, WI) by incubating them with different inducers. For the CYP1A1 and CYP1A2 activity assay, undifferentiated hiPSC, primary hepatocytes and differentiated HLCs were incubated with basal medium containing 50 µM Omeprazole solution (Sigma), or DMSO (0.1%) for 72 hours. Undifferentiated hiPSC, primary hepatocytes and differentiated HLCs were incubated with basal medium containing 20 µM Rifampicin solution (Sigma), or DMSO (0.1%) for 72 hours for the CYP3A4 and CYP3A7 activity assay. Moreover, for the CYP2B6 and CYP2C9 activity assay, undifferentiated hiPSC, primary hepatocytes and differentiated HLCs were incubated with basal medium containing 1000 µM Phenobarbital solution (Sigma), or DMSO (0.1%) for 72 hours. The activity of each enzyme was measured by reading the luminescence using a luminometer (SpectraMax i3x – Molecular Devices) according to the manufacturer’s instructions. All the experiments were performed in triplicate.

### Indocyanin green uptake and release assay

After terminal differentiation HLCs were allow to adhere in a matrigel coated chamber slide. After monolayer formation, incubation of fully differentiated hEB with indocyanin green (IGC, Sigma-Aldrich) was done in basal medium for 1 hour at 37 °C according to the manufacturer’s instructions. With light microscopy using an Olympus IX81, the ICG uptake was initially detected. Then 6 hours later, IGC release was detected to ensure that all the positive cells released the IGC.

### Oil red staining

The cells were tested for the lipid vesicle storage using Oil Red O staining after differentiation, according to the manufacturer’s protocol (abcam – ab150678). We fixed the monolayer of HLCs with 4% paraformaldehyde for 1 hour, and then incubated them for 2 minutes with Propylene Glycol followed by a 6 minute incubation with Oil Red O solution. Following the staining, 1 minute incubation with 85% Propylene Glycol was performed followed by 2 washes with dH_2_O. Staining was detected with light microscopy using an Olympus IX81.

### Periodic Acid-Schiff (PAS) staining

According to the manufacturer instructions (Sigma-Aldrich), the glycogen storage of differentiated hEBs was evaluated using PAS staining. Clusters were initially allowed to adhere to a matrigel coated plate and then after monolayer formation were fixed with 4% paraformaldehyde for 1 hour, then oxidized for 5 minutes with Periodic Acid solution and then washed several times. Following the washes, incubation with Shiff Reagent for 15 minutes was performed followed by color development with dH_2_O for 5 minutes. The staining was then detected with light microscopy using an Olympus IX81.

### Uptake of Low-Density Lipoproteins (LDL) assay

After completion of the differentiation protocol using Dil-Ac-LDL (Alfa Aesar – J65597), LDL uptake assay was performed following the manufacturer instruction. HLCs were allowed to adhere on a matrigel coated plate, and after monolayer formation they were incubated overnight in serum free pre-incubation media containing 0.1% BSA. The differentiated hEBs were incubated the following day for 5 hours at 37 °C with Dil-Ac-LDL 10 µg/mL in pre-incubation media. Then, after the incubation the cells were washed several times with pre-incubation media and fixed with 4% paraformaldehyde for 1 hour. We then performed DAPI staining for the nuclei after fixation for 1 hour at RT. Finally, fluorescent images were acquired with confocal microscopy using an Olympus IX81.

### N- and O-glycan preparation

Approximately 5 × 10^6^ cells were used as starting material Cells were lysed and homogenized in a TRIS (25 mM), NaCl (150 mM), EDTA (5 mM) buffer containing 0.5% CHAPS (w/v), pH 7.4. After overnight dialysis at 4 °C against 50 mM ammonium bicarbonate (NH_4_)HCO_3_); Sigma-Aldrich) buffer the cell homogenates were reduced with a 0.6 M TRIS pH 8.5 solution containing 2 mg/ml of DTT (1,4-Dithiothreitol, Sigma-Aldrich, St Louis, MO), incubating 2 h at 50 °C, and then alkylated with a 0.6 M TRIS pH 8.5 solution containing 12 mg/ml IAA (Iodoacetamide, Sigma-Aldrich), incubating 2 h at RT in the dark. The samples were then dialyzed overnight at 4 °C against 50 mM NH_4_)HCO_3_ before being lyophilized. The samples were next resuspended in 1 ml of 50 mM NH_4_)HCO_3_ solution containing 500 µg/ml TPCK-treated trypsin (Sigma-Aldrich) and incubated at 37 °C overnight. The tryptic digested materials were purified over a C18 Sep-Pak (200 mg) column (Waters, Milford, MA) conditioned with 1 column volume (CV) of methanol (Sigma-Aldrich), 1 CV of 5% of acetic acid, 1 CV of 1-propanol (Sigma-Aldrich), and 1 CV of 5% of acetic acid. The columns were washed with 5% acetic acid (Fisherbrand, Waltham, MA) and the peptides eluted with 2 ml of 20% 1-propanol followed by 2 ml of 40% 1-propanol and then 2 ml of 100% 1-propanol. Eluted fractions were pooled and lyophilized. The dried materials were resuspended in a 200 µl of 50 mM NH_4_)HCO_3_ solution to which 3 µl of PNGaseF (New England Biolabs, Ipswich, MA) was added for a 4h-incubation at 37 °C followed by another 3 µl of PNGaseF for overnight incubation at 37 °C. The N-glycans were purified over a C18 Sep-Pak (200 mg) column conditioned with 1 CV of methanol, 1 CV of 5% of acetic acid, 1 CV of 1-propanol and 1 CV of 5% of acetic acid. The columns were washed with 6 ml of 5% of acetic acid. The flow-through and the washes, containing the N-glycans, were pooled and lyophilized. The N-glycan-released peptides were eluted with 2 ml of 20% 1-propanol followed by 2 ml of 40% 1-propanol and then 2 ml of 100% 1-propanol. The fractions were pooled and lyophilized. The O-glycans were released by reductive beta-elimination by resuspending the dried peptides in 400 µl of a 0.1 M NaOH solution containing 22 mg of NaBH_4_ (Sigma-Aldrich) and incubating at 45 °C overnight. The reaction was stopped with drop-wise addition of pure acetic acid until fizzing stopped. The samples were next loaded onto a Dowex 50 W X8 resin prior conditioned with 4 M HCl, washed with MilliQ water and store in 5% acetic acid. The columns were then washed with 4 ml 5% acetic acid. The flow-through and the washes were collected, pooled and lyophilized. To the dried samples, 1 ml of acetic acid: methanol (1:9, v/v = 10%) was added and proceed to dry under a stream of nitrogen. This co-evaporation step was repeated three more times prior to resuspend the samples in 200 µl of a 50% methanol solution and to load the samples onto a C18 Sep-Pak (200 mg) column conditioned with 1 CV of methanol, 1 CV of 5% of acetic acid, 1 CV of 1-propanol and 1 CV of 5% of acetic acid. The columns were washed with 4 ml of 5% of acetic acid and the free O-glycans contained in the flow-through and wash fractions were collected, pooled and lyophilized.

### N- and O-glycan permethylation

Lyophilized N- and O-glycan samples were incubated with 1 ml of a DMSO (Dimethyl Sulfoxide; Sigma)-NaOH (Sigma-Aldrich) slurry solution and 500 µl of methyl iodide (Sigma-Aldrich) for 30 min under vigorous shacking at RT. The reaction was stopped with 1 ml of MilliQ water and 1 ml of Chloroform (Sigma) was added to purify out the permethylated glycans. 3 ml of Milli-Q water were then added and the mixture briefly vortexed to wash the chloroform fraction. The water was separated by centrifugation and discarded. This wash step was repeated two more times and the chloroform fraction was finally dried before being re-dissolved in 200 ml of 50% methanol prior to be loaded onto a C18 Sep-Pak (50 mg) column conditioned with 1 CV of methanol, 1 CV of MilliQ water, 1 CV of acetonitrile (Sigma) and 1 CV of Milli-Q Water. The columns were washed with 3 ml of 15% acetonitrile and then eluted with 3 ml of 50% acetonitrile. The eluted fraction was lyophilized and then re-dissolved in 10 µl of 75% methanol from which 1 µl was mixed with 1 µl DHB (2,5-dihydroxybenzoic acid (Sigma-Aldrich) (5 mg/ml in 50% acetonitrile with 0.1% trifluoroacetic acid (Sigma-Aldrich) and spotted on a MALDI polished steel target plate (Bruker Daltonics, Bremen, Germany).

### Mass spectrometry of N- and O-glycans and data acquisition and analyses

MS data was acquired on a Bruker UltraFlex II MAL DI-TOF Mass Spectrometer instrument. Reflective positive mode was used and data was recorded between 500 m/z and 6000 m/z for the N-glycans and between 0 m/z and 4000 m/z for the O-glycans. For each MS N- or O-glycan profiles, the aggregation of at least 20,000 laser shots were considered for data extraction. MS signals of a signal/noise ratio of at least 2 were considered and only MS signals matching an N- or O-glycan composition were considered for further analysis. Subsequent MS post-data acquisition analyses were made using mMass^91^.

### Cell transplantation

The Institutional Animal Care and Use Committee (IACUC) approved the use of animals for experimentation in this study. Through intraperitoneal injection of 950 mg kg^−1^ of sterile D-galactosamine dissolved in Hanks Balanced Salt Solution (Sigma-Aldrich), acute liver failure was induced in 270–350 g athymic nude rats (Crl:NIH-*Foxn1*^*rnu*^, Charles River Laboratories, Wilmington, MA). Then under inhalational anaesthesia, 80–100 hiPSC-EB-HLCs were injected into the spleen body through the caudal pole of the spleen. The caudal pole was ligated following injection. Experimental groups consisted of animals transplanted with the hiPSC-EB-HLCs with HAMEC and hiPSC-EB-HLCs without HAMEC. Animals receiving differentiation medium only constituted our negative control. The healthy controls consisted of animals without liver injury transplanted with hiPSC-EBs. Animals were monitored daily and received standard chow and water ad libitum. The animals’ survival was tracked as a primary end point. They were sacrificed either after 14 days or earlier if they had moribund appearance or greater than 30% body weight loss in accordance with predefined humane care criteria. All of our experiments were conducted in accordance with the approved IACUC guidelines.

### Serum analysis

The tail vein was phlebotomized prior to transplantation, 48–72 hours after transplantation, and at time of sacrifice. We measured of serum alanine transaminase (ALT) concentration in whole blood using VetScan 2.0 (Abaxis, Union City, CA). Presence of human albumin in the rat serum was then evaluated using a Human Albumin ELISA Quantitation Set that was non-cross reactive with rat albumin (Bethyl Laboratories).

### Histology and immunohistochemistry

The animals’ liver and spleen samples were recovered at sacrifice or death followed by fixation using 10% neutral buffered formalin. Subsequently, the cells were embedded in paraffin, sectioned with hematoxylin and eosin staining for histologic assessment.

Using xylene-substitute and ethanol, the paraffin-embedded slides were deparaffinized and immunohistochemistry was performed on rat liver and spleen sections in order to identify the presence of human albumin and human platelet EC adhesion molecule (PECAM)−1. Endogenous peroxidase activity was blocked with 4% hydrogen peroxide following deparaffinization.

Regarding human albumin detection and (PECAM)−1, non-specific binding was blocked with 2% donkey serum for 60 minutes (Sigma-Aldrich), then the slides were incubated with a non-cross reactive goat antibody to human albumin primary antibody (Bethyl Laboratories; A80-129; 1:500) and a non–crossreactive mouse antibody to human platelet EC adhesion molecule (PECAM)−1 (Santa Cruz; sc-133091) for 60 minutes. We then used the secondary antibody of HRP-conjugated donkey antibody to goat IgG (Santa Cruz; 1:200) and a goat antibody to mouse IgG1-HRP (AbD Serotec, Raleigh, NC) was used as the secondary antibody for 60 minutes. Metal-enhanced diaminobenzidine substrate was used to activate the horseradish peroxidase in both cases (Thermo-Scientific, Waltham, MA). The slides were counterstained with Mayer’s haematoxylin (Sigma-Aldrich).

For immunofluorescence staining of the rat liver and spleen sections, we fixed the slides with 4% (wt/vol) paraformaldehyde for 30 minutes; then they were permeabilized using 0.3% (vol/vol) Triton-X 100 in PBS for 30 minutes and blocked with 0.5% (vol/vol) goat serum (Sigma-Aldrich) in PBS for 1 hr RT. The samples were then incubated with the primary antibody at 4 °C overnight. Following several washes, samples were then incubated with the secondary antibody at room temperature for 1 hour. We used the following human specific primary antibodies: non-cross reactive goat antibody to human albumin primary antibody (Bethyl Laboratories; 1:500), mouse anti human HNF-3β (RY-7) (Santa Cruz, sc-101060; 1:100) and rabbit anti human C-MET (Santa Cruz, sc-10; 1:100) and non-cross-reactive mouse antibody to human platelet EC adhesion molecule (PECAM)-1 (Santa Cruz).

### Statistical analysis

All the experiments performed in our results were run in triplicate or more from independent experiments. Quantitative data are reported as mean ± standard deviation. Comparisons were conducted using Student t tests or analysis of variance for continuous variables, and Fisher’s exact test or Chi-square tests for categorical variables. All the statistical analyses were performed using JMP 9.0 (Stata Corp LP, College Station, TX).

### Statement of institutional and/or licensing committee and use of human tissue samples

All methods and experimental protocols were approved and carried out in accordance with relevant guidelines and regulations of both Virginia Commonwealth University and Beth Israel Deaconess Medical Center from the respective Institutional Committees.

All human liver tissues used in this study had direct approval by the Virginia Commonwealth University (VCU) - Health System IRB (approval number 00124) and were prepared in accordance with the FDA/IND (number 13909) following all requirements for informed consent and regulatory guidelines. The Hume Lee Transplant Center Tissue Bank of VCU Health System, Richmond, VA is also acknowledged. Cryopreserved human hepatocytes were used in accordance with both institutions guidelines.

### Ethical statement

All experimental protocols using both hiPSCs and animal studies were approved by the Institutional Animal Care and Use Committee (IACUC protocol number 003-2016 and Beth Israel Deaconess Medical Center has Animal Welfare Assurance #D16-00093 (A3153-01)) and performed following the policy of both Virginia Commonwealth University and Beth Israel Deaconess Medical Center. The Animal Research Facility (ARF) assists investigators in their obligation to plan and conduct animal experiments in accord with the highest scientific, humane and ethical principles. This is achieved by development and maintenance of a comprehensive, high quality animal care program, which is AAALAC accredited and complies with all Federal, State and Local laws. All the animal protocols were approved by the IACUC.

## Supplementary information

Supplementary info
