# Supplementary material for: Generation of fully functional hepatocyte-like organoids from human induced pluripotent stem cells mixed with Endothelial Cells
Source: Sci Rep. 2019 Jun 20;9:8920. doi: 10.1038/s41598-019-45514-3 (PMC6586904; doi:10.1038/s41598-019-45514-3)
Supplement: Supplementary file 1 — Supplementary info [file 41598_2019_45514_MOESM1_ESM.docx]

**Generation of fully functional hepatocyte-like organoids from human induced pluripotent stem cells mixed with Endothelial Cells**

Giuseppe Pettinato^1^,* Sylvain Lehoux^1,2^, Rajesh Ramanathan^3^, Mohamed M. Salem^1,5^, Li-Xia He^1^, Oluwatoyosi Muse^6^, Robert Flaumenhaft^6^, Melissa T. Thompson^1^, Emily A. Rouse^2^, Richard D. Cummings^1^, Xuejun Wen^4^, Robert A. Fisher^1^,*

**Supplementary information**

**
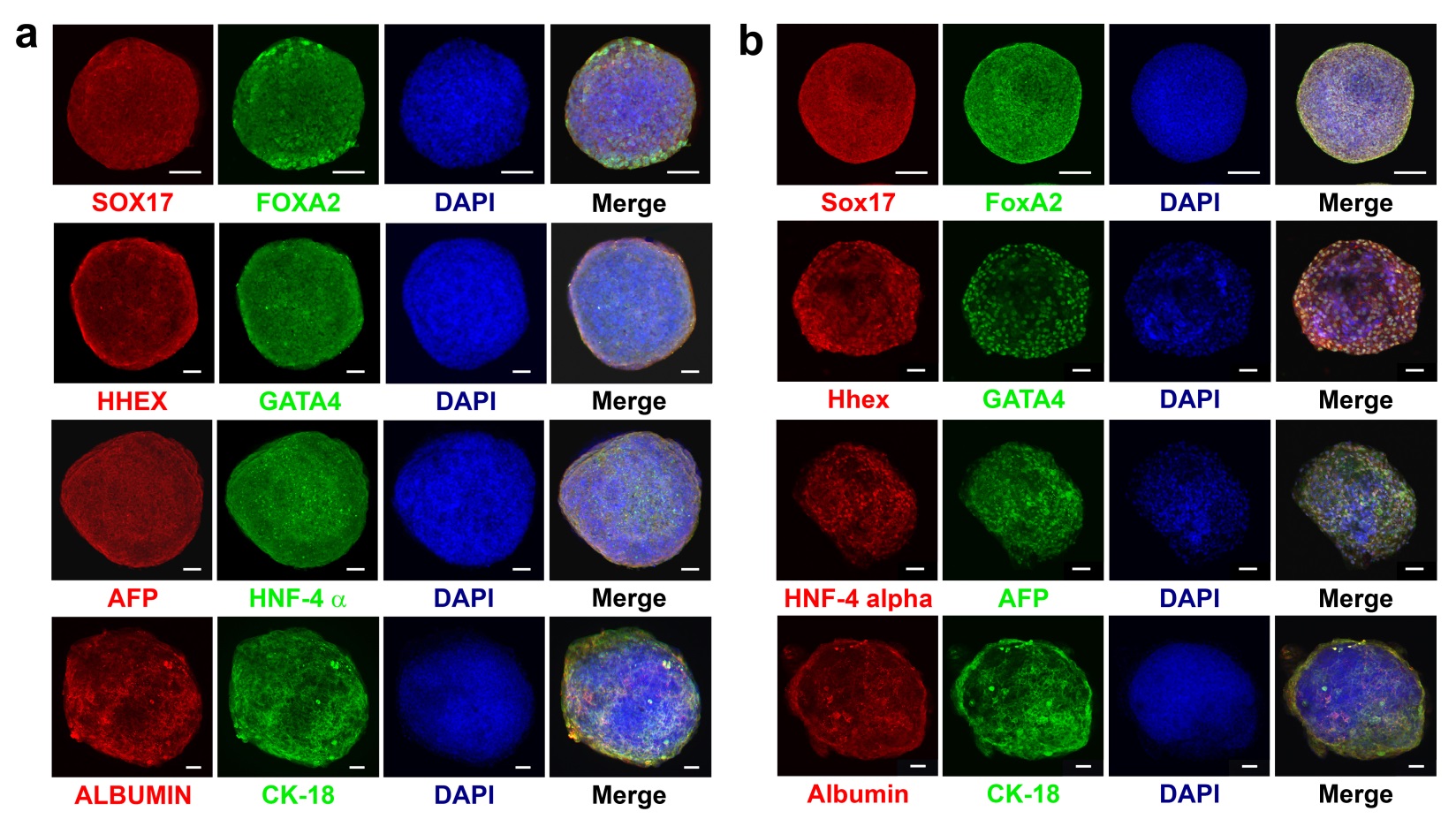
**

**Supplementary Figure 1:** Immunofluorescence analysis of stage-specific markers. The stage-specific protein expressions analysis during the differentiation process showed a similar pattern between the two experimental conditions hiPSC-EB-HLCs (**a**) and hiPSC-EB+EC-HLCs (**b**), indicating that the presence of HAMEC did not affected the progression of the differentiation process. The markers studied were: SOX17 and FOXA2 for the definitive endoderm; HHEX and GATA4 for the foregut endoderm; AFP and HNF-4αfor the hepatic progenitor cells; and ALBUMIN and CK-18 for the mature. DAPI stains for cell nuclei. Scale bar 100 µm.

**
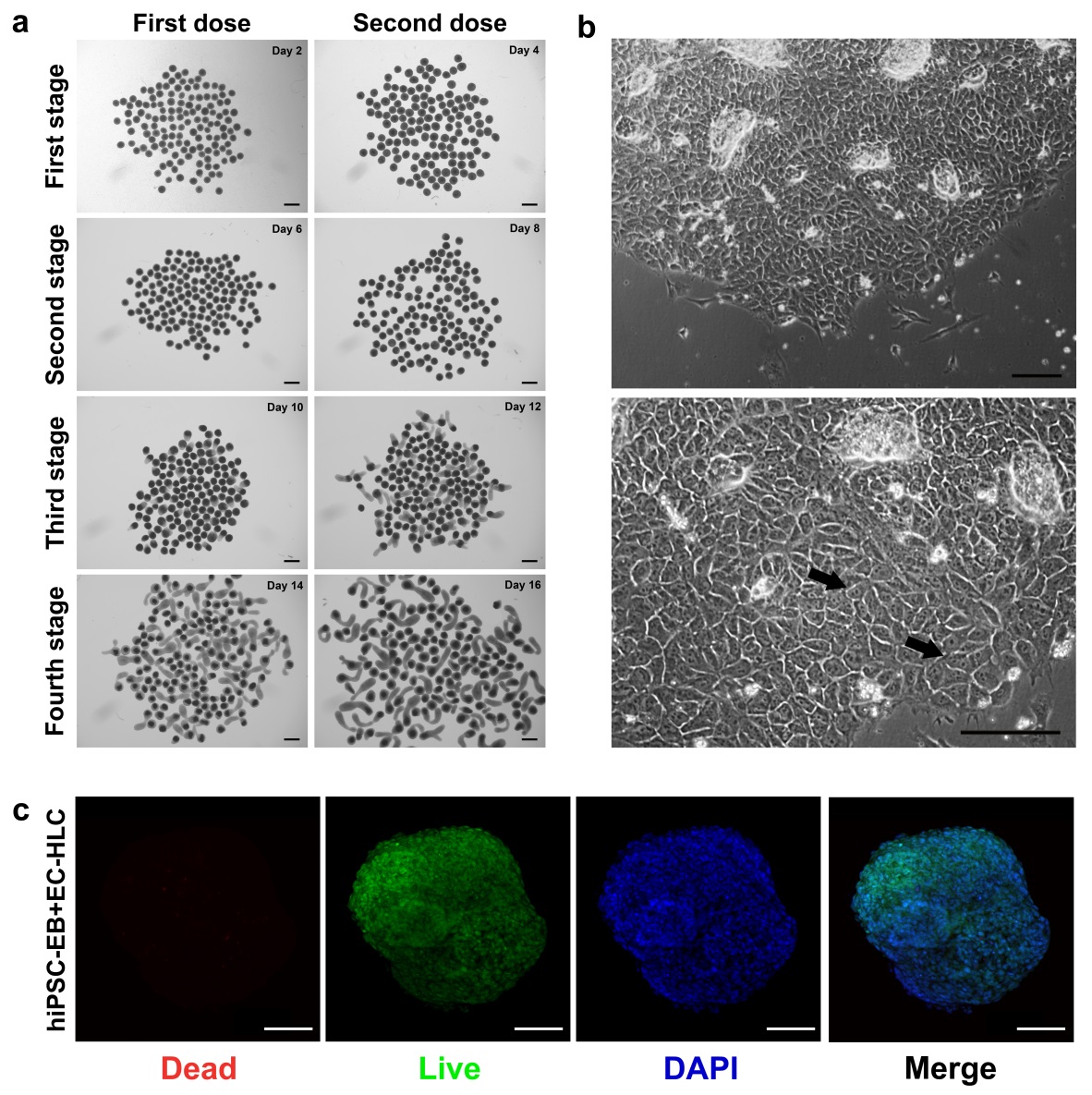
**

**Supplementary figure 2:** Morphology study of differentiated HLCs. (**a**) The clusters showed a progressive change of morphology during the differentiation process from the initial dose of the first stage, through the end of the differentiation protocol. In particular, the hEBs kept a nearly spherical morphology until the end of the second stage, to then start to develop extrusions at the beginning of the third stage, that lead to the formation of elongated structures containing a net of canaliculi within the terminally differentiated HLCs. Scale bar 1 mm (**b**) Light microscopy for the morphological assessment of both hiPSC-EB-HLCs with and without HAMEC displayed the presence of a polygonal shape with enriched cytoplasmic granules within the cells after differentiation (arrows). Upper 10X magnification; lower 20X magnification. Scale bar 100 µm. (**c**) The hiPSC-EB-HLCs with and without HAMEC increased their size from approximately 500 μm after 24 hours of their formation to 800-1,000 μm at the end of differentiation process without any core necrosis at any time. The picture shows a live-dead stain of a representative hiPSC-EB+EC-HLC at the end of the differentiation process. Scale bar 200 μm.

**
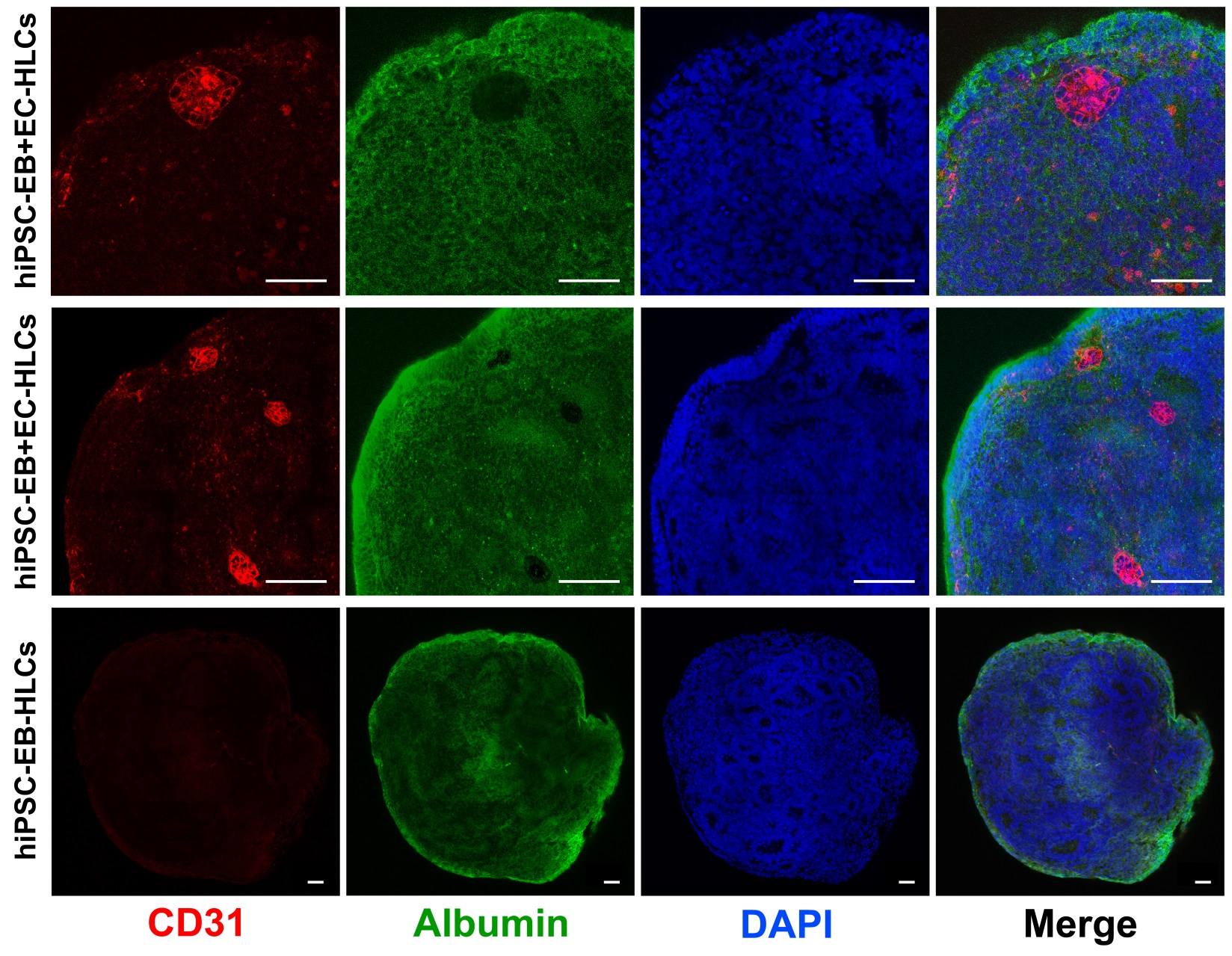
**

**Supplementary figure 3:**  Demonstrative microscopic optical cross-section views at the end of the differentiation process displaying the ration between HAMEC CD31 positive cells (about 15% - red) and differentiated HLCs albumin positive cells (about 89% - green). On the bottom figure overview of differentiated HLCs without HAMEC did not show any CD31 positive staining. Scale bar 50 μm

**
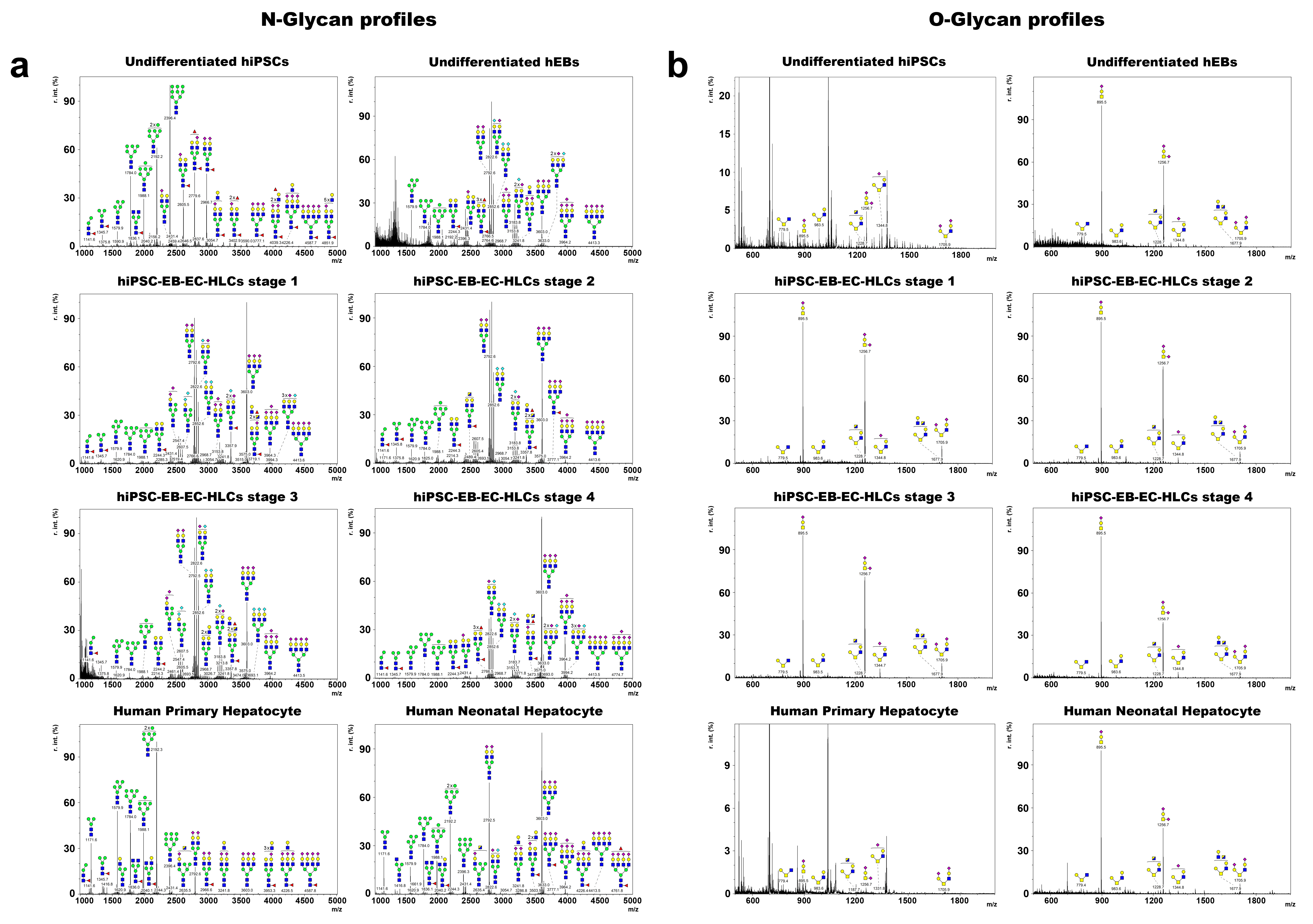
 Supplementary Figure 4:** MALDI-ToF mass spectrometry (**A**) N-glycans and (**B**) O-glycans profiles. Partial annotations (for clarity) of MS N-glycans profiles. Masse/charge (m/z). Mannose ( ), Galactose ( ), N-acetylglucosamine ( ), fucose ( ), N-acetylneuraminic acid ( ), and N-glycolylneuraminic acid ( ). Glycan sequences are predicted based on known rules of N- and O-glycan biosynthesis and compositional analyses.

**
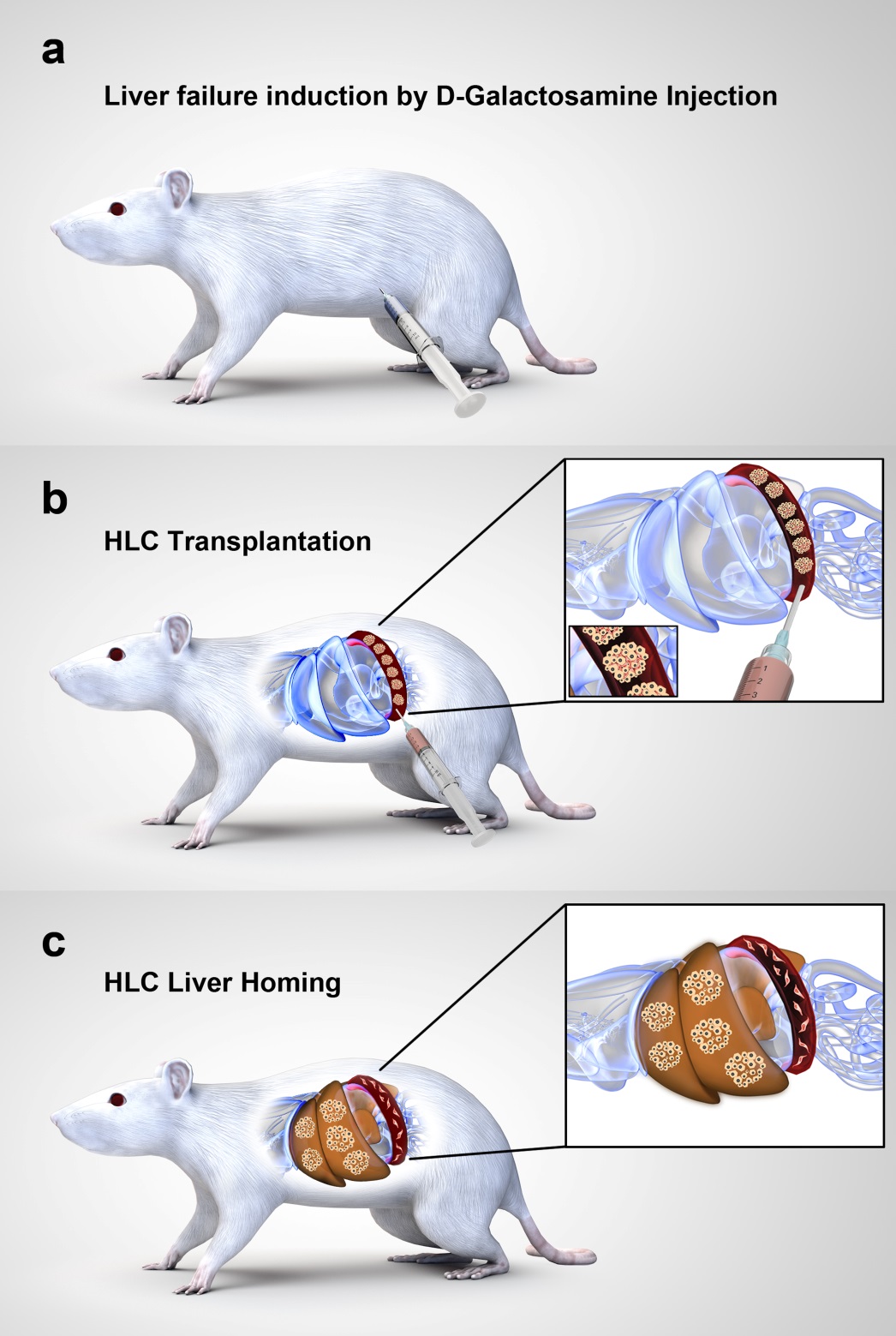
**

**Supplementary Figure 5:** Transplantation of HLCs in a rat model. Schematic representation of the acute liver failure animal model used for the transplantation of our differentiated HLCs. (**A**) Liver failure induction through intraperitoneal D-Galactosamine injection; (**B**) Transplantation of HLCs into the caudal pole of the rat’ spleen; (**C**) Spontaneous migration of the HLCs to the rat’ liver and while the HAMEC remained into the rat’ spleen.

**
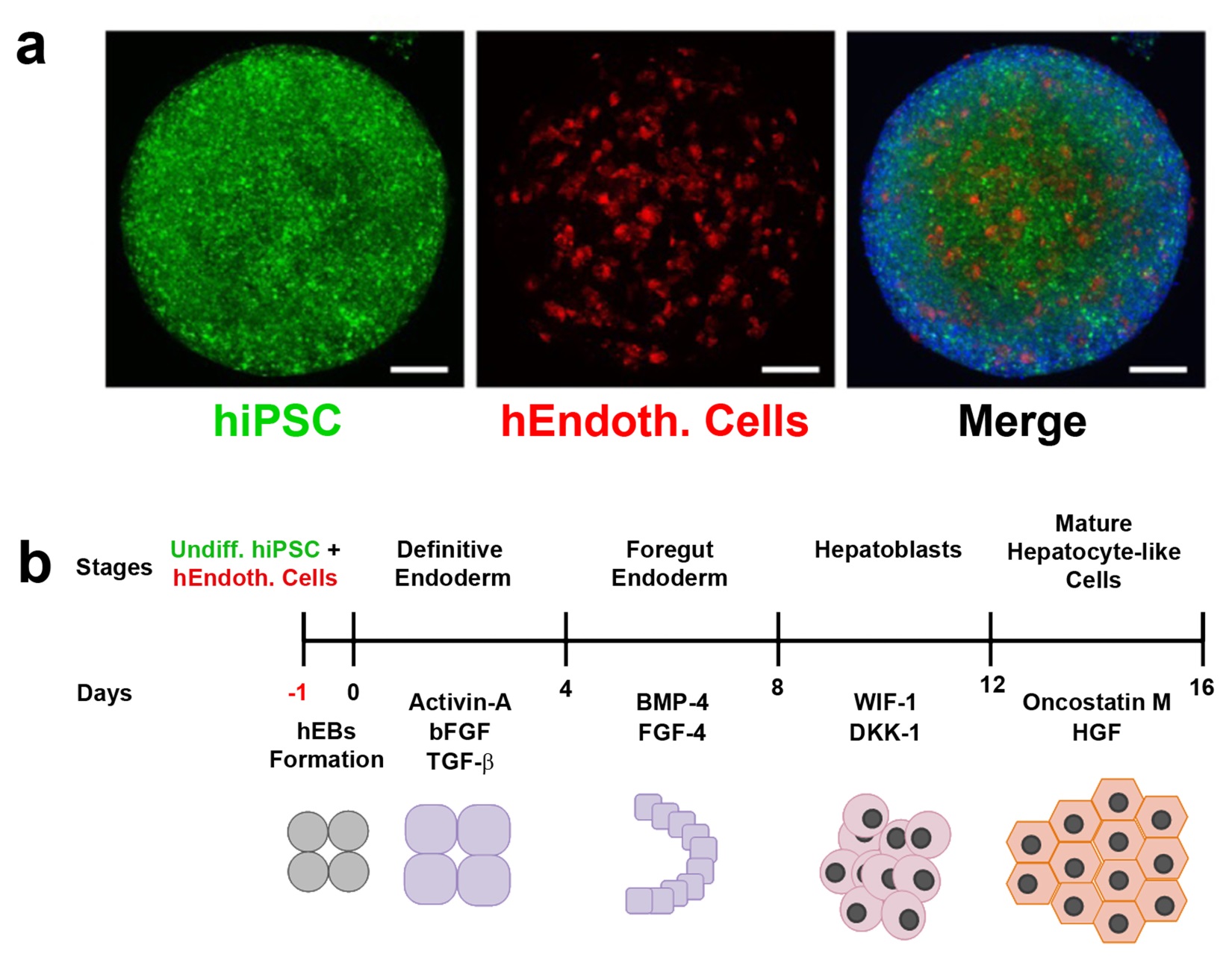
**

**Supplementary Figure 6:** Formation of hEBs with hiPSCs interlaced with HAMEC. (**A**) Immunofluorescent image representing a hiPSC EB+ECs immediately after extraction from the agarose mold; Scale bar 100 µm. (**B**) Simplified representation of the 4-stages differentiation protocol showing the main growth factors used at each stage: the protocol mimics the embryologic developmental stages of the liver; beginning from the human induced pluripotent stem cells (hiPSCs), the cells progress to Definitive Endoderm (DE), going towards Foregut Endoderm (FE) and then to Hepatic Progenitor Cells (HPCs) or Hepatoblasts. The last maturation stage drives the cells towards mature Hepatocyte-Like Cells (HLCs).

**
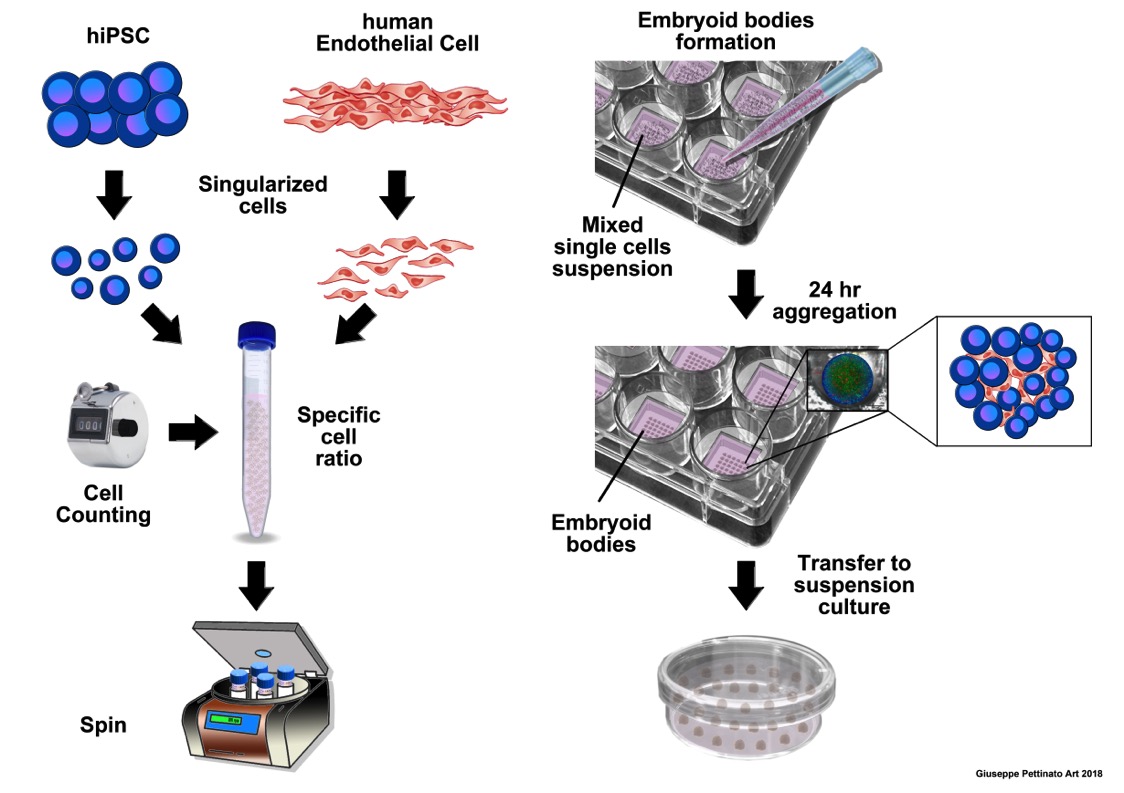
**

**Supplementary figure 7:** Schematic representation of the work-flow for the hiPSC-EB+ECs formation using our 3-D agarose micromold technology. Briefly, both hiPSCs and HAMEC were dissociated into single cells separately and counted after centrifugation. Both types of cells were mixed into the same tube with a ratio of 3:1 (hiPSC-hECs) and after mixing the cells thoroughly, they were plated into our agarose micromold for the hEBs formation. 24 hours after, the hiPSC-EB+ECs were transferred in suspension culture for the application of the differentiation protocol.
